# Wearable Inertial Phenotyping of Freezing of Gait in Parkinson’s Disease: Biomechanical Signatures, Subject-Independent Classification, and Clinical Associations

**DOI:** 10.3390/bioengineering13101150

**Published:** 2026-10-02

**Authors:** Augusto Ielo, Lilla Bonanno, Angelo Quartarone, Serena Dattola

**Affiliations:** IRCCS Centro Neurolesi Bonino-Pulejo, S.S. 113 Via Palermo, C. da Casazza, 98124 Messina, Italy; augusto.ielo@irccsme.it (A.I.); angelo.quartarone@irccsme.it (A.Q.); serena.dattola@irccsme.it (S.D.)

**Keywords:** Parkinson’s disease, freezing of gait, wearable sensors, inertial measurement units, machine learning, deep learning, digital biomarkers

## Abstract

Freezing of gait (FoG) in Parkinson’s disease is clinically heterogeneous, yet wearable-sensor studies predominantly focus on binary FoG detection. We investigated whether right-ankle inertial signals can characterize clinically meaningful FoG manifestations within a three-stage conditional framework. Accelerometer and gyroscope data from 22 participants in the FoG-STAR dataset were segmented into 1 s windows. Three subject-independent classification stages were evaluated separately: locomotor non-FoG versus FoG, kinetic versus akinetic FoG, and shuffling versus trembling. Nine interpretable biomechanical features were compared with Logistic Regression, RBF-SVM, Random Forest, and a raw temporal 1D-CNN using frozen participant-independent partitions. The CNN yielded a mean Stage-1 balanced accuracy (0.877 ± 0.034), whereas for Stage-2 kinetic–akinetic discrimination, Logistic Regression and the CNN yielded mean balanced accuracies of 0.730 ± 0.044 and 0.727 ± 0.065, respectively. Stage-3 performance was lower and more variable (Logistic Regression, 0.666 ± 0.133). Nested leave-one-subject-out analysis restricted to mixed-phenotype participants further highlighted subject-level heterogeneity. Kinetic FoG showed greater residual movement amplitude and movement-rate characteristics than akinesia. Protocol-observed locomotor FoG burden was positively associated with FoG-Q (ρ=0.536) and MDS-UPDRS III (ρ=0.785). Wearable inertial signals therefore showed clinically interpretable differences among FoG manifestations, while finer phenotype discrimination remained heterogeneous and requires external validation in independent cohorts.

## 1. Introduction

Freezing of gait (FoG) is a brief, episodic failure to generate effective forward stepping despite the intention to walk and represents one of the most disabling gait disturbances in Parkinson’s disease (PD) [1]. Although FoG can occur earlier in the disease course, its frequency generally increases with disease progression and greater motor impairment [2]. Clinically, FoG is closely linked to postural instability and falls, with important consequences for mobility, independence, and injury risk [3]. Its impact also extends beyond gait performance itself: individuals with FoG report substantial restrictions in daily function and poorer quality of life, even when other aspects of disease severity are considered [4]. These features make FoG a clinically important but difficult phenomenon to quantify objectively and reproducibly.

FoG is also heterogeneous. Episodes may be provoked by gait initiation, turning, narrow passages, destination approach, dual-tasking, or other situations that increase locomotor demand, and the observable motor expression is not uniform. Classical clinical descriptions distinguish shuffling with very small ineffective steps, trembling of the legs in place, and complete akinesia, with different manifestations potentially occurring in different episodes or sequentially within the same episode [5]. This variability has motivated models in which FoG is considered an episodic breakdown emerging from interactions among motor, cognitive, and perceptual processes rather than a single stereotyped motor deficit [6]. The resulting clinical complexity also contributes to the variable response of FoG to medication, cueing, and rehabilitation strategies [7]. At the signal level, trembling manifestations have been associated with characteristic high-frequency lower-limb oscillations, particularly within the approximately 3–8 Hz range, with evidence that these oscillations propagate from distal to more proximal segments during freezing [8]. Together, these observations support the view that clinically defined FoG manifestations may contain distinct and potentially measurable biomechanical signatures.

The episodic and context-dependent nature of FoG limits assessment based exclusively on retrospective questionnaires or brief clinical observation and has stimulated extensive work on wearable sensing. Early inertial studies demonstrated that lower-limb acceleration can capture frequency-domain changes during FoG and introduced quantitative measures based on the relative increase of power in a “freeze” band [9]. Subsequent research expanded the use of accelerometers, gyroscopes, and multi-sensor systems for automatic detection in laboratory and home-like conditions. A systematic review of wearable FoG and fall detection highlighted both the promise of these systems and the substantial heterogeneity in sensor placement, protocols, sample sizes, and validation strategies [10]. More recently, an international state-of-the-art review emphasized that robust FoG measurement requires explicit attention to annotation procedures, testing context, validation design, and the distinction between clinical events and algorithmic outputs [11]. These methodological considerations are particularly important when wearable measurements are intended not only to detect whether FoG is present, but also to characterize how it is expressed.

Machine-learning approaches have progressively shifted FoG analysis from fixed signal thresholds toward multivariate and learned temporal representations. Deep-learning models applied to inertial data have demonstrated that discriminative temporal patterns can be learned directly from wearable signals, including during home monitoring [12], while subject-independent machine-learning systems have shown that wearable signals can support automatic FoG quantification during FoG-provoking protocols [13]. Nevertheless, the dominant formulation in the literature remains binary FoG detection or FoG prediction. Reviews of wearable-sensor methods have repeatedly identified limited cohort sizes, strong inter-individual variability, class imbalance, and inconsistent subject-independent validation as major obstacles to generalizable deployment [14]. A more recent review reached similar conclusions despite the growing number and apparent performance of published models [15]. Importantly, cross-dataset evaluation has shown that models with strong within-dataset performance can lose substantial accuracy when transferred to different cohorts and acquisition settings, underscoring the need to separate representation capacity from genuine subject-independent generalization [16].

Although wearable FoG research has predominantly emphasized binary detection, automatic phenotype characterization is not entirely new. Wearable systems have previously been used to distinguish shuffling from trembling manifestations [17], and more recent approaches have extended automatic assessment to trembling, akinetic, and, in some settings, shuffling FoG [18,19]. These studies establish the feasibility of automated FoG subtyping, but generally formulate the problem as direct manifestation detection or multiclass labeling. The present study addresses a different question: whether clinically meaningful FoG phenotyping can be decomposed into conditional subject-independent decisions and, at each level, whether a compact biomechanically interpretable representation is sufficient or whether learned temporal representations provide additional discriminative value. Several related questions therefore remain open. First, it is unclear to what extent clinically meaningful FoG manifestations can be characterized using interpretable biomechanical features rather than relying exclusively on learned temporal representations. Second, shuffling, trembling, and complete akinesia form a clinically meaningful but imbalanced set of manifestations. This clinical organization motivates a conditional formulation in which progressively finer phenotype distinctions are evaluated only within the relevant parent state. Third, aggregate window-level performance may not reflect the more stringent scenario in which a model must discriminate phenotypes within an unseen participant who actually exhibits both target manifestations. Finally, wearable-derived FoG quantities should be related cautiously to clinical severity: daily-life monitoring, structured provocation tests, and self-report capture overlapping but non-equivalent aspects of FoG [20].

The FoG-STAR dataset provides an opportunity to address these issues because it combines synchronized multi-site inertial recordings with continuous activity labels, expert FoG annotations, manifestation-level labels for shuffling, trembling, and akinesia, and clinical assessments in participants with PD [21]. Work based on the same recording cohort has previously examined binary FoG detection across sensor types, body positions, motor activities, datasets, and machine-learning models [22]. In the present study, locomotor FoG recognition therefore serves as the first reference node of a broader phenotyping framework rather than as the primary methodological novelty. The availability of both FoG and non-FoG context, together with manifestation-specific annotations, enables a clinically structured analysis of progressively finer FoG distinctions. Thus, the novelty lies in the conditional organization and comparative evaluation of the phenotype decisions rather than in introducing a new binary FoG detector or a new manifestation taxonomy.

Accordingly, the present study formulated wearable FoG phenotyping as a clinically aligned hierarchical classification framework composed of three conditional subject-independent classification stages: (i) locomotor non-FoG versus locomotor FoG; (ii) given FoG, kinetic versus akinetic FoG, where kinetic FoG comprised shuffling and trembling; and (iii) given kinetic FoG, shuffling versus trembling. Each stage was evaluated independently on the clinically eligible ground-truth samples rather than as an end-to-end decision cascade. The contribution of the study is therefore not the first automated recognition of FoG manifestations, but their conditional evaluation in terms of subject-independent discriminability, biomechanical interpretability, representation-learning requirements, and participant-level clinical associations. The study had four objectives: to characterize interpretable right-ankle inertial signatures of the annotated FoG manifestations; to compare conventional feature-based models with a compact raw temporal convolutional neural network under frozen subject-independent splits; to examine phenotype discrimination in participants who exhibited both target classes using nested leave-one-subject-out sensitivity analysis; and to investigate associations between protocol-observed digital FoG phenotypes and clinical measures. This design was intended to clarify which levels of FoG characterization are captured by interpretable biomechanics, where learned temporal representations add value, and where inter-participant heterogeneity remains the principal limitation.

## 2. Materials and Methods

### 2.1. Study Design and Data Source

This study was a secondary analysis of the publicly available FoG-STAR (Freezing of Gait Severity, Tasks, Activities, and Ratings) dataset. The dataset is openly archived in Zenodo (DOI: 10.5281/zenodo.17838806) and is accompanied by a dedicated data descriptor published in Scientific Data [21,23]. No new participants were recruited and no new sensor, clinical, or behavioral data were collected for the present study; all analyses were performed on previously acquired data distributed as part of the FoG-STAR resource.

The original FoG-STAR acquisition was conducted during a structured motor protocol designed to elicit or challenge freezing of gait in people with Parkinson’s disease. The protocol comprised seven tasks: the Timed Up-and-Go test, standing for 1 min, walking back and forth, walking through a doorway, walking while carrying a glass of water, walking while counting backward, and a 360° turning task. According to the original study protocol, assessments were performed in the medication “Off” state. Not all participants completed every task, particularly the more demanding motor and dual-task conditions; therefore, the amount and composition of protocol exposure varied across participants [21].

### 2.2. Participants and Clinical Variables

The FoG-STAR cohort comprised 22 individuals with idiopathic Parkinson’s disease diagnosed according to the Movement Disorder Society clinical diagnostic criteria [24]. In the original data collection, participants were recruited from two Italian university movement-disorders clinics. The source study excluded individuals with dementia or with significant musculoskeletal, cardiovascular, psychiatric, or other neurological conditions that could materially affect gait. For the present secondary analysis, all 22 participants were retained at the cohort level; no additional exclusion criterion was applied on the basis of demographic or clinical variables. Eligibility for the individual classification stages was determined solely by the availability of the clinically relevant annotated states and contexts, as described in Section 2.4. Participants in whom no FoG was observed during the structured protocol were retained where eligible and are referred to throughout as “participants with no FoG observed during the protocol”, rather than as clinical non-freezers.

The clinical data file was linked to the sensor recordings through the participant identifier and contained age, sex (stored under the variable name gender in the source dataset), disease duration, Hoehn and Yahr stage, MDS-UPDRS Part III, FoG-Q, Montreal Cognitive Assessment (MoCA), Falls Efficacy Scale–International (FES-I), and Parkinson’s Disease Questionnaire–8 (PDQ-8) scores. Disease stage was represented by the Hoehn and Yahr scale [25], while motor impairment was quantified using Part III of the Movement Disorder Society-sponsored revision of the Unified Parkinson’s Disease Rating Scale (MDS-UPDRS III) [26]. FoG-related self-reported severity was represented by the Freezing of Gait Questionnaire (FoG-Q) [27]. Global cognitive status was assessed using the MoCA [28]; concerns related to falling were assessed using the FES-I [29]; and health-related quality of life was represented by the PDQ-8 [30].

Clinical information was complete for age, sex, MDS-UPDRS III, FoG-Q, and PDQ-8 in all 22 participants. Disease duration, Hoehn and Yahr stage, MoCA, and FES-I each contained one missing observation (n=21 available for each variable). Missing clinical values were not imputed. Consequently, analyses involving clinical variables were performed using the available complete participant pairs for the variables under consideration, as detailed in Section 2.10.

### 2.3. Inertial Sensing and FoG Annotations

The FoG-STAR acquisition used a wireless body area network comprising four inertial measurement units (IMUs; Nordic Thingy:52, Nordic Semiconductor). One IMU was positioned on the wrist of the most affected side, two were placed on the lower legs immediately above the right and left ankles, and one was positioned on the lower back at approximately the L3–L5 level [21]. Each device incorporated an MPU-9250 inertial sensor (InvenSense/TDK Corporation, San Jose, CA, USA) and provided tri-axial acceleration and tri-axial angular-velocity signals. The released dataset contains these six inertial channels for each of the four sensor locations, yielding 24 sensor channels in total. Signals were sampled at a nominal frequency of 60 Hz; acceleration and angular velocity were expressed in *g* and degrees/s, respectively.

Sensor recordings were temporally aligned with synchronized video and accompanied by sample-level contextual annotations [21]. For each sample, the released sensor table provides activity, FoG occurrence, FoG manifestation, participant, session, and task identifiers. The activity annotation distinguishes walking, sitting, standing, sit-to-stand, stand-to-sit, right turning, and left turning. FoG annotations were derived from synchronized video review by two neurologists with expertise in Parkinson’s disease and movement disorders. FoG onset was defined at the last effective step preceding the freezing interval and FoG offset at the first effective step following it; disagreements between raters were resolved by consensus. Within annotated FoG intervals, three observable manifestations were retained: shuffling, trembling in place, and complete akinesia. Although the released data field is named fog_severity, in the present study these labels were treated as FoG manifestations rather than as a patient-level clinical severity scale, because different manifestations can occur sequentially within the same FoG episode.

The present analysis used the right-ankle IMU as the primary sensing location. This choice provided a distal lower-limb measurement directly related to stepping dynamics while maintaining a single fixed sensor configuration across all analyses. In the released data, all six right-ankle accelerometer and gyroscope channels were complete for the FoG-labeled samples used to define the manifestation classes. For analyses that also included non-FoG data, windows containing incomplete right-ankle samples were excluded according to the completeness procedure described in Section 2.5. The resulting input therefore consisted of six synchronized right-ankle channels—three acceleration and three angular-velocity axes—sampled at 60 Hz.

### 2.4. Clinically Aligned Hierarchical FoG Phenotyping Framework

We formulated FoG phenotyping as a clinically aligned hierarchical classification framework composed of three conditional subject-independent classification stages (Figure 1). The hierarchy was used to decompose the problem into progressively finer clinical distinctions rather than to implement an end-to-end decision cascade. Accordingly, “hierarchical” refers here to the clinical nesting of the target definitions, not to sequential propagation of model predictions. Each stage was evaluated independently on the clinically eligible ground-truth samples to characterize its intrinsic subject-independent discriminability. Consequently, predictions generated at one stage were never propagated to a subsequent stage, and an error at an upstream stage could not alter the test set or target labels of a downstream stage.

Stage 1 addressed FoG recognition within a strictly locomotor context. Samples were eligible only when the annotated activity corresponded to walking, right turning, or left turning. Within this subset, all samples annotated as FoG—irrespective of manifestation—formed the positive class, whereas locomotor samples not annotated as FoG formed the negative class. Restricting Stage 1 to these activities prevented non-locomotor states from entering the FoG-versus-non-FoG comparison and ensured that the first decision reflected locomotor FoG recognition.

Stage 2 addressed the principal manifestation-level distinction conditional on ground-truth FoG. All annotated FoG samples were eligible, without imposing the locomotor restriction used in Stage 1. Shuffling and trembling were grouped as kinetic FoG, because clinically observable lower-limb movement persisted during these manifestations, whereas complete akinesia constituted akinetic FoG. This grouping is conceptually consistent with the updated International Consortium for Freezing of Gait framework, which identifies the presence or absence of observable lower-limb movement as the primary distinction between kinetic and akinetic FoG [31]. The original FoG-STAR manifestation labels were nevertheless preserved: the dataset’s shuffling category was not relabeled as the broader consensus category of kinetic-no-trembling FoG.

Stage 3 further characterized only the ground-truth kinetic-FoG subset. Samples annotated as shuffling were contrasted with samples annotated as trembling in place, again using all annotated activity contexts represented in the dataset. Stage 3 therefore evaluated whether the two original kinetic manifestations in FoG-STAR could be distinguished after conditioning on the presence of kinetic FoG. This final stage retained the dataset’s original manifestation taxonomy and did not introduce additional phenotype labels.

The resulting framework can be summarized as follows: Stage 1, locomotor non-FoG versus locomotor FoG; Stage 2, conditional on FoG, kinetic versus akinetic FoG; and Stage 3, conditional on kinetic FoG, shuffling versus trembling. Stage-specific eligibility was determined exclusively from the ground-truth activity and FoG annotations before model fitting. All three stages were subsequently evaluated using participant-independent data partitions, as detailed in Section 2.5. This design allowed the classification difficulty of each clinically meaningful decision to be quantified separately without claiming that hierarchical routing improves flat multiclass classification.

### 2.5. Signal Segmentation, Window Generation, and Frozen Subject-Independent Splits

Raw sensor records were processed in acquisition order as reconstructed from the source participant, session, task, and timestamp information. Temporal continuity was assessed from the native 60 Hz time base. The median positive inter-sample interval was approximately 16.7 ms, and a temporal discontinuity was defined using a gap threshold of 2.5 nominal sampling intervals (approximately 41.7 ms). Contiguous homogeneous segments were then constructed such that no segment crossed a participant, session, task, activity, temporal-discontinuity, or FoG-state boundary. The state-specific segment pools comprised locomotor non-FoG, shuffling, trembling, and complete akinesia. This segmentation was performed before stage-specific classification labels were assigned, thereby preserving the original manifestation boundaries.

All primary analyses used fixed-length 1 s windows, corresponding to 60 samples at the native sampling frequency. For the deterministic reference dataset, non-overlapping windows were generated independently within each homogeneous segment using a reproducible random temporal phase. Specifically, for a segment containing *N* samples, the maximum number of complete windows was K=⌊N/60⌋, leaving a residual of R=N−60K samples. A single deterministically seeded integer offset was drawn uniformly from 0,…,R, and the *K* windows were then placed at consecutive 60-sample intervals from that offset. This procedure avoided systematically anchoring every reference window to the onset of an annotated segment while guaranteeing that no reference window crossed a segment boundary.

Right-ankle sensor completeness was evaluated only after the deterministic reference windows had been defined. A reference window was retained only when all six right-ankle accelerometer and gyroscope channels contained finite values for all 60 samples. This ordering was fixed to ensure that missing-data boundaries did not redefine the temporal phase or placement of otherwise valid reference windows. The resulting deterministic windows constituted the common reference manifest used for biomechanical feature extraction and for all validation and outer-test evaluations. Validation and test sets retained their naturally occurring class distributions and were never downsampled or rebalanced.

A single five-fold subject-independent partition was fixed before model fitting and reused across all three stages and all model families. The common outer test sets were: fold 1, participants 5, 7, 12, 15, and 16; fold 2, participants 1, 8, 13, 17, and 22; fold 3, participants 4, 6, 19, and 20; fold 4, participants 3, 9, 11, and 14; and fold 5, participants 2, 10, 18, and 21. The fold assignment was selected at the participant level using a seeded balancing procedure before any classifier was evaluated, with the aim of maintaining representation of the relevant FoG states and mixed-phenotype participants across folds; no model prediction or outer-test performance metric entered the fold construction. Within each outer-training partition, a fixed participant-independent validation subset was also defined and reused across models: participants 3, 9, 10, and 19 for fold 1; 9, 10, 11, and 19 for fold 2; 1, 9, 10, and 15 for fold 3; 7, 12, 13, and 19 for fold 4; and 4, 7, 13, and 19 for fold 5. All remaining eligible participants formed the corresponding inner-training set. Thus, no participant contributed samples to more than one of the training, validation, or test partitions within a given outer fold.

For the feature-based models, training, validation, and test data were obtained from the same deterministic 1 s reference manifest, with any weighting or balancing restricted to the inner-training data. For the raw temporal convolutional neural network, training was intentionally separated from deterministic evaluation: each stage-relevant homogeneous segment was additionally divided into contiguous right-ankle-complete blocks, and valid 60-sample start positions within those blocks were retained as a dynamic training pool. During model fitting, windows could therefore be resampled dynamically from inner-training subjects only, whereas validation and outer-test performance was always computed on the unchanged deterministic reference windows. This separation allowed stochastic training augmentation without altering the composition, temporal phase, or natural class distribution of the evaluation sets.

### 2.6. Interpretable Biomechanical Features

Nine interpretable features were extracted from each deterministic 1 s right-ankle window to characterize complementary aspects of movement amplitude, rate of change, and frequency content. Let $a\left[n\right]={\left[a_{x}\left[n\right],a_{y}\left[n\right],a_{z}\left[n\right]\right]}^{⊤}$ denote tri-axial acceleration and $\omega \left[n\right]={\left[\omega_{x}\left[n\right],\omega_{y}\left[n\right],\omega_{z}\left[n\right]\right]}^{⊤}$ tri-axial angular velocity at sample *n*, with sampling frequency $f_{s}=60$ Hz. Their vector magnitudes were defined as $m_{a}\left[n\right]={∥a\left[n\right]∥}_{2}$ and $m_{\omega }\left[n\right]={∥\omega \left[n\right]∥}_{2}$. Four time-domain features were computed: the population standard deviation of $m_{a}$, the root-mean-square (RMS) magnitude of the acceleration jerk vector, the population standard deviation of $m_{\omega }$, and the RMS magnitude of the angular-acceleration vector. Acceleration jerk and angular acceleration were obtained from first differences of the corresponding tri-axial signals multiplied by $f_{s}$:

(1) $$j\left[n\right]=f_{s}\left(a[n]-a[n-1]\right),\;\alpha \left[n\right]=f_{s}\left(\omega [n]-\omega [n-1]\right),$$

and their vector RMS values were computed as

(2) $${\mathrm{RMS}}_{j}=\sqrt{\frac{1}{N-1}\sum_{n=1}^{N-1}{∥j\left[n\right]∥}_{2}^{2}},\;{\mathrm{RMS}}_{\alpha }=\sqrt{\frac{1}{N-1}\sum_{n=1}^{N-1}{∥\alpha \left[n\right]∥}_{2}^{2}}.$$

These four measures constituted the temporal feature subset used in the subsequent sensitivity analyses.

Five frequency-domain features were derived from the acceleration and angular-velocity magnitudes. Before spectral analysis, the mean was removed from each magnitude signal and the resulting 60-sample sequence was multiplied by a Hann window. A one-sided discrete Fourier transform was then obtained using the real-valued fast Fourier transform, and spectral power was calculated from the squared Fourier magnitude. Spectral summaries were restricted to the 0.5–15 Hz analysis band. Motivated by the frequency ranges historically associated with FoG-related lower-limb oscillations [8,9], low-frequency power was defined over 0.5≤f<3 Hz and freeze-band power over 3≤f<8 Hz.

For both acceleration and angular velocity, relative freeze-band power was defined as

(3) $$P_{\mathrm{rel},3-8}=\frac{P_{3-8}}{P_{0.5-15}},$$

where $P_{3-8}$ denotes summed spectral power in the 3–8 Hz band and $P_{0.5-15}$ denotes summed power in the full analysis band. For acceleration, a logarithmic freeze index was additionally computed as

(4) $$FI_{\log}=\log_{10}\left(\frac{P_{3-8}+\varepsilon }{P_{0.5-3}+\varepsilon }\right),$$

where ε is a small positive numerical constant preventing division by zero. Because the freeze index is a ratio rather than an absolute measure of 3–8 Hz signal amplitude, its value can increase either when freeze-band power increases or when low-frequency power decreases. Accordingly, its direction need not track overall movement amplitude across different FoG manifestations. Finally, normalized spectral entropy was computed separately for acceleration and angular velocity within the 0.5–15 Hz analysis band. If $p_{k}$ denotes the spectral power at the *k*-th included frequency bin and $q_{k}=p_{k}/\sum_{j}p_{j}$, entropy was defined as

(5) $$H_{\mathrm{spec}}=-\frac{\sum_{k=1}^{M}q_{k}\log\left(q_{k}\right)}{\log(M)},$$

with zero-power terms omitted from the logarithmic sum. The five spectral features were therefore acceleration relative 3–8 Hz power, acceleration logarithmic freeze index, acceleration spectral entropy, angular-velocity relative 3–8 Hz power, and angular-velocity spectral entropy. Together with the four temporal measures, these formed the fixed nine-feature representation used by the traditional machine-learning models.

Biomechanical characterization was performed descriptively at the participant level to prevent participants contributing longer recordings from disproportionately influencing the summaries. For each participant and motor state, the median of each window-level feature was first calculated. Group-level state summaries were then expressed as the median and interquartile range of these participant-level values. Within-participant phenotype contrasts were additionally calculated only in participants who exhibited both states relevant to the comparison. For Stage 2, each participant’s median kinetic-FoG value, pooling shuffling and trembling windows, was contrasted with that participant’s median akinetic-FoG value. For Stage 3, the participant-level median during shuffling was contrasted with the corresponding median during trembling. These paired contrasts were summarized by their median difference and by the number of participants showing positive or negative differences. They were treated as descriptive biomechanical analyses; no hypothesis-test *p*-values were calculated.

### 2.7. Traditional Machine-Learning Benchmark

The fixed nine-feature representation described in Section 2.6 was evaluated using three conventional supervised classifiers: L2-regularized Logistic Regression, a support-vector machine with a radial basis function (RBF) kernel, and Random Forest. Logistic Regression was retained as the primary interpretable feature-based baseline, whereas RBF-SVM and Random Forest were included as secondary nonlinear benchmarks to determine whether nonlinear decision boundaries provided a consistent advantage over the linear model. The RBF-SVM and Random Forest candidate grids were fixed before inspection of the corresponding outer-test results. Support-vector machines and Random Forests follow the formulations introduced by Cortes and Vapnik [32] and Breiman [33], respectively.

All models used the same deterministic 1 s windows, the same nine input features, and the frozen participant-independent training, validation, and test partitions described in Section 2.5. Class and manifestation imbalance was handled only within the inner-training data through a subject-aware weighting scheme. For each stage, a target total weight was first assigned to each underlying manifestation leaf. In Stage 1, locomotor non-FoG received a total target mass of 0.50, while shuffling, trembling, and akinesia each received 1/6, yielding an overall FoG target mass of 0.50. In Stage 2, shuffling and trembling each received 0.25 and akinesia 0.50. In Stage 3, shuffling and trembling each received 0.50. Within each leaf, the assigned mass was divided equally among the inner-training participants exhibiting that leaf and subsequently divided equally among the windows contributed by that participant to that leaf. The resulting window weights were finally normalized to have a mean of one. This procedure prevented participants with longer recordings from dominating either a class or a manifestation-specific leaf.

For Logistic Regression and RBF-SVM, feature standardization was estimated exclusively from the weighted inner-training data. For feature *j*, the weighted mean and variance were

(6) $$\mu_{j}=\frac{\sum_{i=1}^{n}w_{i}x_{ij}}{\sum_{i=1}^{n}w_{i}},\;\sigma_{j}^{2}=\frac{\sum_{i=1}^{n}w_{i}{\left(x_{ij}-\mu_{j}\right)}^{2}}{\sum_{i=1}^{n}w_{i}},$$

and the same $\mu_{j}$ and $\sigma_{j}$ were then applied unchanged to the validation and test partitions. Random Forest was fitted to the unstandardized feature values because tree-based splits do not require feature scaling. The same subject-aware sample weights were supplied during fitting of all three classifier families.

Logistic Regression used an L2 penalty and the liblinear solver, with a maximum of 5000 iterations. The regularization parameter was selected from

C∈{0.01,0.1,1,10,100}.

The RBF-SVM grid comprised the 16 combinations

C∈{0.1,1,10,100},γ∈{scale,0.01,0.1,1}.

The Random Forest benchmark used 500 trees with the Gini splitting criterion, bootstrap sampling, and the Cartesian product

max_depth∈{6,12,None},

min_samples_leaf∈{5,1},max_features∈{sqrt,1.0},

for a total of 12 candidate configurations. Fixed random states were used for Logistic Regression and Random Forest; the SVM optimization was deterministic for the specified data and hyperparameters.

Hyperparameter selection was performed independently within each outer fold and stage using only the frozen participant-independent validation partition. Candidate configurations were ranked first by validation balanced accuracy and then, in the event of a numerical tie, by validation macro-F1. Remaining ties were resolved by the fixed candidate order; for Logistic Regression this favored the smaller *C*, while the Random Forest order prioritized more regularized, simpler trees. Once the hyperparameters had been selected, the final model for that fold was fitted again on the same inner-training partition only; the validation participants were not merged back into the fitting set. The outer-test partition remained completely unused during preprocessing, weighting, hyperparameter selection, and model fitting.

Balanced accuracy was the primary performance metric because the target distributions were unequal and varied across stages and held-out participants. Macro-F1, class-specific recall, and receiver-operating-characteristic area under the curve (ROC-AUC) were reported as secondary metrics. Logistic Regression used a fixed probability threshold of 0.5 for hard classification; no threshold optimization was performed. For the RBF-SVM, class predictions were obtained directly from the fitted decision function and its continuous decision scores were used for ROC-AUC calculation. Random Forest class probabilities were used for ROC-AUC and the classifier’s default class decision was used for hard predictions. Performance was first calculated separately in each natural-distribution outer-test fold and then summarized as the mean and standard deviation across the five participant-independent folds. No hypothesis-test *p*-values were used to compare the traditional classifiers.

### 2.8. Raw Temporal Convolutional Neural Network

To complement the interpretable feature-based models, a compact one-dimensional convolutional neural network (1D-CNN) was evaluated directly on the raw right-ankle inertial signals. Each input consisted of a 1 s window with dimensions 60×6, containing 60 consecutive samples from the three accelerometer and three gyroscope axes. The architecture was fixed before interpretation of the outer-test results and no architecture search was performed. The network comprised a first 1D convolution from 6 to 32 channels with kernel size 5 and padding 2, followed by a rectified linear unit (ReLU); a second 1D convolution from 32 to 64 channels with kernel size 5 and padding 2, followed by ReLU; adaptive global average pooling over the temporal dimension; dropout with probability 0.20; and a single linear output unit. The model contained 11,361 trainable parameters.

Signal normalization was estimated separately within each outer fold and classification stage using only the corresponding inner-training subjects. Channel-wise means and variances were computed from the stage-relevant, right-ankle-complete training blocks using subject-balanced moments: each participant contributed equally to the estimated first and second moments regardless of recording duration. The resulting six channel means and standard deviations were applied unchanged to dynamically sampled training windows and to the deterministic validation and outer-test windows. No validation- or test-subject samples contributed to normalization.

CNN training used the dynamic block-based sampling pool described in Section 2.5. At each draw, a manifestation leaf was first sampled according to fixed stage-specific probabilities. For Stage 1, locomotor non-FoG was assigned probability 0.50, while shuffling, trembling, and akinesia were each assigned 1/6; for Stage 2, shuffling and trembling were each assigned 0.25 and akinesia 0.50; and for Stage 3, shuffling and trembling were each assigned 0.50. Conditional on the selected leaf, a participant exhibiting that leaf was sampled uniformly. One of that participant’s eligible contiguous blocks was then selected with probability proportional to the number of valid 60-sample start positions in the block, after which a valid start position was sampled uniformly within the selected block. This hierarchical sampler therefore balanced the target classes while preventing participants with longer recordings from dominating training. Validation and test windows were never dynamically resampled or rebalanced.

Optimization used AdamW [34] with a learning rate of $10^{-3}$ and weight decay of $10^{-4}$. The loss function was BCEWithLogitsLoss, with no additional class weighting because imbalance was addressed through the training sampler. Mini-batch size was 64, and 1024 dynamically sampled windows were generated per epoch. Training was limited to 40 epochs. After each epoch, performance was evaluated on the fixed participant-independent validation set using a probability threshold of 0.5. The model state with the highest validation balanced accuracy was retained; validation macro-F1 was used as a tie-breaker when balanced accuracy was numerically identical. Early stopping was triggered after seven consecutive epochs without improvement according to this criterion.

To quantify training stochasticity while preserving the same participant-level evaluation design, each stage and outer fold was trained independently with five random seeds. Each seed produced a separately early-stopped model using the same inner-training and validation participants. For the primary CNN estimate, predicted probabilities for each outer-test window were averaged across the five seed-specific models within that fold. Hard predictions were then obtained from the ensemble probability using the fixed threshold of 0.5, with no threshold retuning. Balanced accuracy was the primary metric, while macro-F1, class-specific recall, and ROC-AUC were secondary metrics, consistent with the traditional benchmark. Final performance was summarized as the mean and standard deviation across the five frozen subject-independent outer folds after seed-level probability averaging.

An additional Stage 1 activity-context sensitivity analysis was performed using the frozen outer-test predictions from all four classifiers. No model was retrained or retuned for this analysis. Stage 1 predictions were stratified according to the ground-truth locomotor activity context as walking, right turning, or left turning, and balanced accuracy, class-specific recall, and ROC-AUC were recomputed separately within each activity stratum when both classes were represented. Activity labels were used only for this post-prediction stratification and were never provided as classifier inputs. An activity-equal descriptive balanced accuracy was additionally calculated as the unweighted mean of the valid within-activity balanced accuracies, preventing the relative frequency of walking and turning windows from determining this summary.

### 2.9. Mixed-Subject Nested Leave-One-Subject-Out Sensitivity Analysis

A complementary sensitivity analysis was performed to examine phenotype discrimination under the more stringent condition in which each held-out participant exhibited both target classes. This analysis was restricted to Stage 2 and Stage 3, because its purpose was to assess within-participant generalization of FoG phenotype discrimination rather than FoG recognition itself. A participant was defined as mixed for a stage when at least one deterministic 1 s reference window was available for each of the two corresponding target classes. Eight participants met this criterion for Stage 2 (kinetic and akinetic FoG), contributing 878 reference windows in total, whereas five participants met it for Stage 3 (shuffling and trembling), contributing 112 windows. These mixed-subject counts are distinct from the participant counts in the main stage-wise benchmark, which included 22, 16, and 15 participants in Stages 1, 2, and 3, respectively. Participants contributing to the main Stage 3 benchmark were not required to exhibit both shuffling and trembling within the same individual; the mixed-subject nested LOSO sensitivity analysis imposed this additional within-participant eligibility criterion, which reduced the eligible Stage 3 subset from 15 to 5 participants.

Three predefined feature representations were evaluated using L2-regularized Logistic Regression: the four-feature temporal subset, the five-feature spectral subset, and the combined nine-feature representation described in Section 2.6. The classifier specification, subject-aware weighting principle, weighted feature standardization, fixed probability threshold of 0.5, and candidate regularization values were kept consistent with the primary interpretable benchmark.

For each stage and feature set, an outer leave-one-subject-out (LOSO) loop was applied to the mixed-participant subset. At each outer iteration, all windows from one mixed participant were reserved as the test set and all remaining mixed participants formed the outer-training pool. Hyperparameter selection was then performed entirely within that outer-training pool using an inner LOSO procedure. For each candidate value of *C*, every remaining participant was held out once as the inner-validation subject, while the classifier was fitted on the other participants. Subject-aware training weights and weighted standardization parameters were recomputed using only the participants available in each inner-training split and then applied unchanged to the corresponding inner-validation participant.

Candidate *C* values were ranked by the subject-equal mean inner-validation balanced accuracy across the inner LOSO iterations. Mean macro-F1 was used as the first tie-breaker and the smaller *C* was preferred if a tie remained. After selection, Logistic Regression was refitted on all participants in the outer-training pool using newly estimated subject-aware weights and weighted standardization parameters, and was then evaluated once on the completely held-out mixed participant. No information from the outer-test participant entered feature scaling, weighting, hyperparameter selection, or model fitting.

Balanced accuracy was the primary endpoint because both target classes were present in every outer-test participant. Macro-F1, class-specific recall, and ROC-AUC were calculated as secondary metrics. Since one participant constituted each outer test fold, final performance was summarized as the unweighted mean and standard deviation across held-out participants, giving each participant equal contribution irrespective of the number of available windows. The natural within-participant class distribution was retained during testing. No hypothesis-test *p*-values were calculated.

This nested LOSO analysis was treated as a sensitivity analysis rather than as a replacement for the main frozen five-fold benchmark. In particular, the Stage 3 temporal-subset result was interpreted as exploratory because only five independent participants exhibited both shuffling and trembling, and it was not used post hoc to redefine the primary combined-feature model.

### 2.10. Clinical Digital Phenotype and Statistical Analysis

Participant-level digital FoG phenotype measures were derived directly from the sample-level ground-truth annotations in the released FoG-STAR recordings and were independent of classifier predictions. Four complementary measures were considered. First, protocol-observed locomotor FoG burden was defined using only samples annotated as walking, right turning, or left turning:

(7) $$\mathrm{FoG}\;{\mathrm{burden}}_{\mathrm{loc}}=100\times \frac{N_{\mathrm{FoG},\mathrm{loc}}}{N_{\mathrm{all},\mathrm{loc}}},$$

where $N_{\mathrm{FoG},\mathrm{loc}}$ denotes the number of locomotor samples annotated as FoG and $N_{\mathrm{all},\mathrm{loc}}$ denotes the total number of samples observed in the same locomotor contexts. This measure was calculated for all 22 participants; participants with no FoG observed during the structured protocol therefore had a value of zero. Because the denominator depended on the amount of locomotor activity completed during the protocol, locomotor exposure duration was also summarized for each participant. The resulting quantity was interpreted strictly as protocol-observed locomotor FoG burden and not as an estimate of daily-life FoG prevalence.

Second, among participants with protocol-observed FoG, the akinetic fraction of FoG was defined as

(8) $$\mathrm{Akinetic}\;\mathrm{fraction}=100\times \frac{N_{\mathrm{akinesia}}}{N_{\mathrm{shuffling}}+N_{\mathrm{trembling}}+N_{\mathrm{akinesia}}}.$$

Third, among participants exhibiting kinetic FoG, the trembling fraction of kinetic FoG was calculated as

(9) $$\mathrm{Trembling}\;{\mathrm{fraction}}_{\mathrm{kinetic}}=100\times \frac{N_{\mathrm{trembling}}}{N_{\mathrm{shuffling}}+N_{\mathrm{trembling}}}.$$

Finally, manifestation diversity was summarized exploratorily using normalized Shannon entropy across shuffling, trembling, and akinesia. If $p_{m}$ denotes the proportion of all FoG samples assigned to manifestation *m*, normalized manifestation entropy was

(10) $$H_{\mathrm{manifestation}}=-\frac{\sum_{m=1}^{3}p_{m}\log\left(p_{m}\right)}{\log(3)},$$

with zero-probability terms omitted. This quantity ranges from 0, when the observed FoG is entirely represented by a single manifestation, to 1, when the three manifestations are equally represented.

Clinical associations were evaluated at the participant level using Spearman rank correlation coefficients. Three associations were designated as primary: protocol-observed locomotor FoG burden with FoG-Q, protocol-observed locomotor FoG burden with MDS-UPDRS III, and akinetic fraction of FoG with FoG-Q. All remaining associations between the digital FoG phenotype measures and disease duration, Hoehn and Yahr stage, MDS-UPDRS III, FoG-Q, MoCA, FES-I, and PDQ-8 were considered exploratory. Age and sex were used for cohort characterization and were not included as clinical association endpoints.

Uncertainty around each Spearman coefficient was quantified using a nonparametric participant-level bootstrap [35]. For each association, 10,000 bootstrap samples were generated by resampling participants with replacement from the complete participant pairs available for that analysis. Spearman’s ρ was recalculated in each bootstrap sample, and the 2.5th and 97.5th percentiles of the bootstrap distribution were reported as the 95% confidence interval. Missing clinical observations were handled by available-case analysis, without imputation, so the effective sample size was reported for each association.

Given the small cohort and the exploratory nature of several clinical comparisons, no hypothesis-test *p*-values were calculated and no multiplicity-adjusted significance testing was performed. Associations were interpreted according to their direction, magnitude, and bootstrap uncertainty rather than by dichotomous significance thresholds. In particular, confidence intervals excluding zero were not treated as proof of clinical validity or causality. All digital phenotype measures reflected observations obtained during the structured FoG-provoking protocol and were therefore interpreted within that acquisition context. An additional exposure-adjusted sensitivity analysis was performed for the two primary associations involving protocol-observed locomotor FoG burden. Partial Spearman correlations were calculated while controlling for participant-level locomotor exposure duration. Burden, the corresponding clinical variable, and exposure duration were first transformed to average ranks. Ranked burden and ranked clinical scores were then separately residualized with respect to ranked exposure duration using ordinary least squares with an intercept, and the resulting residuals were correlated. The association between locomotor exposure duration and FoG burden was also summarized using Spearman correlation. Uncertainty for these sensitivity estimates was quantified using 10,000 participant-level bootstrap resamples, with the complete correlation procedure recomputed within each resample. The original unadjusted associations remained the primary analyses.

### 2.11. Software, Reproducibility, and Analysis Lock

All analyses were implemented in Python 3.12.12. Data management and numerical processing were performed primarily with NumPy (version 2.3.5) and pandas (version 2.3.3); conventional machine-learning models and performance metrics were implemented with scikit-learn (version 1.8.0); statistical calculations used SciPy (version 1.16.3) where applicable; the raw temporal CNN was implemented in PyTorch (version 2.13.0); and analysis figures were generated with Matplotlib (version 3.10.8). The complete workflow was organized as a sequence of analysis steps producing explicit processed-data tables, reports, figures, and machine-readable configuration records.

Reproducibility was enforced at the data-partition and model-evaluation levels. The deterministic 1 s reference-window manifest and the five participant-independent outer folds were generated once and then reused unchanged across the feature-based and CNN benchmarks. Fixed random seeds were used for stochastic procedures, including reference-window phase generation, participant-level fold construction, conventional-model random states where applicable, and the five CNN training repetitions. Preprocessing parameters, subject-aware weights, hyperparameter selection, and early stopping were always estimated without access to the corresponding outer-test participants.

Key analysis specifications were recorded in analysis-lock files before execution of the associated benchmark steps. These records included the fixed feature representation, classifier candidate grids, CNN architecture and optimization parameters, evaluation metrics, probability threshold, and seed-ensemble procedure. After inspection of the frozen benchmark results, the corresponding model specifications were not retuned on the basis of outer-test performance. The mixed-subject nested LOSO analysis and the clinical association analysis were executed as separately defined analyses and did not modify the primary five-fold benchmark.

The final manuscript-level synthesis was generated exclusively from the frozen outputs of the completed analytical pipeline. This synthesis step performed no additional model fitting, hyperparameter optimization, or inferential testing; instead, it verified numerical consistency across the previously generated reports and assembled the manuscript-ready tables and summaries. Accordingly, all numerical results reported in the manuscript can be traced to saved analysis outputs rather than being recomputed manually during manuscript preparation.

Although the participant-independent partitions prevent direct train–test subject overlap, the same frozen outer folds were reused during model development and comparison. The reported cross-validation performance should therefore be interpreted as subject-independent development and benchmarking rather than as untouched external validation. Independent evaluation on a separate cohort remains necessary to establish external generalizability.

## 3. Results

### 3.1. Cohort and FoG Manifestation Distribution

The analysis included 22 participants with Parkinson’s disease. FoG was observed during the structured protocol in 16 participants, whereas 6 participants had no FoG observed during the protocol. Shuffling, trembling, and complete akinesia were observed in 8, 12, and 9 participants, respectively, and 3 participants exhibited all three manifestations. Participant characteristics are summarized in Table 1. Clinical variables were complete for age, sex, MDS-UPDRS III, FoG-Q, and PDQ-8; disease duration, Hoehn and Yahr stage, MoCA, and FES-I each had one missing observation.

The deterministic stage-specific reference sets reflected the conditional structure of the analysis. Stage 1 comprised 3542 one-second windows from all 22 participants, including 2556 locomotor non-FoG windows and 986 locomotor FoG windows. Stage 2 comprised 1004 FoG windows from 16 participants, of which 290 were kinetic FoG and 714 were akinetic FoG. Stage 3 comprised the 290 kinetic-FoG windows from 15 participants, including 83 shuffling and 207 trembling windows. The larger FoG count in Stage 2 than in Stage 1 reflects the fact that Stage 2 retained FoG from all annotated activity contexts, whereas Stage 1 was restricted to walking and turning; 18 akinesia windows occurring outside the Stage 1 locomotor context were therefore retained in Stage 2 but excluded from Stage 1. Participant-level stage-specific window counts are reported in Table S4.

### 3.2. Biomechanical Signatures of FoG Manifestations

Participant-level summaries showed descriptive differences in residual movement across FoG manifestations, with the most consistent contrasts observed between kinetic FoG and complete akinesia (Table 2). Complete akinesia showed the lowest movement-amplitude and movement-rate values among the FoG states. In particular, median acceleration magnitude variability was 0.069 [0.022, 0.105] during akinesia, compared with 0.151 [0.057, 0.192] during shuffling and 0.176 [0.104, 0.274] during trembling. A similar pattern was observed for acceleration jerk, angular-velocity variability, and angular-acceleration RMS. The complete participant-level summary for all nine pre-specified biomechanical features is provided in Table S1. Selected participant-level biomechanical signatures across motor states are illustrated in Figure 2.

The within-participant Stage 2 contrasts supported this pattern. Among the eight participants who exhibited both kinetic and akinetic FoG, the median kinetic-minus-akinetic difference in acceleration jerk RMS was 9.888 and was positive in all eight participants. Angular-velocity magnitude variability showed a median paired difference of 18.585, positive in 7/8 participants, while angular-acceleration RMS showed a median difference of 574.972, also positive in 7/8 participants. Acceleration magnitude variability was higher during kinetic FoG in 7/8 participants, with a median paired difference of 0.114. In contrast, acceleration relative power in the 3–8 Hz band showed a near-zero median paired difference (0.002) with an evenly split direction across participants (4/8 positive and 4/8 negative). The acceleration freeze index was likewise directionally inconsistent, with kinetic-minus-akinetic differences positive in 3/8 participants and negative in 5/8 participants; the median difference was −0.101.

The Stage 3 shuffling-versus-trembling contrasts were more heterogeneous. Angular-velocity magnitude variability was the most directionally consistent feature, with a median shuffling-minus-trembling difference of 12.009 and higher values during shuffling in all five participants exhibiting both manifestations. By contrast, acceleration magnitude variability, acceleration jerk RMS, angular-acceleration RMS, relative 3–8 Hz acceleration power, and acceleration freeze index showed mixed within-participant directions. Thus, the descriptive biomechanical separation between kinetic and akinetic FoG was more consistent for movement-amplitude and movement-rate measures, whereas the finer distinction between shuffling and trembling showed greater inter-participant variability.

### 3.3. Subject-Independent Hierarchical Stage-Wise Classification

Model performance differed across the three conditional classification stages (Table 3). Unless otherwise specified, classification metrics are reported as the mean and standard deviation across the five frozen participant-independent outer folds. These standard deviations describe fold-to-fold variability and should not be interpreted as inferential confidence intervals. Model comparisons across folds were descriptive only, and no claim of statistical superiority was made. Stage 1, locomotor non-FoG versus locomotor FoG, showed the highest overall discriminability. The raw temporal 1D-CNN yielded a mean balanced accuracy of 0.877±0.034, with macro-F1 0.835±0.049 and ROC-AUC 0.934±0.032. Among the traditional models, RBF-SVM yielded a mean Stage 1 balanced accuracy of 0.845±0.032, followed descriptively by Random Forest (0.837±0.044) and Logistic Regression (0.833±0.031). The descriptive difference between the CNN and RBF-SVM was +0.031 in mean balanced accuracy. Across the six participants with no FoG observed during the protocol, the Stage 1 CNN false-positive rate was 0.043±0.021.

The distribution of Stage 1 classes differed across locomotor activities. Walking comprised 1817 non-FoG and 56 FoG windows, whereas right turning comprised 313 non-FoG and 389 FoG windows and left turning comprised 426 non-FoG and 541 FoG windows. To assess whether this contextual imbalance could account for Stage 1 discrimination, the frozen outer-test predictions were examined separately within each activity. For the CNN, balanced accuracy was 0.839 during walking, 0.785 during right turning, and 0.821 during left turning, with corresponding ROC-AUC values of 0.886, 0.815, and 0.869. The activity-equal descriptive balanced accuracy was 0.815. Similar within-activity discrimination was observed for the traditional models, indicating that Stage 1 performance was not confined to contrasts between different locomotor activity categories. Complete activity-stratified results for all four model families are reported in Table S3.

For Stage 2, kinetic versus akinetic FoG, the combined nine-feature Logistic Regression achieved a balanced accuracy of 0.730±0.044, macro-F1 of 0.720±0.057, and ROC-AUC of 0.816±0.053. The raw temporal CNN yielded a mean balanced accuracy of 0.727±0.065, corresponding to a descriptive difference of −0.004 relative to Logistic Regression. RBF-SVM and Random Forest achieved balanced accuracies of 0.724±0.069 and 0.699±0.038, respectively. The corresponding mean values were therefore close across Logistic Regression and the CNN, but the five outer folds were not treated as independent replicates and these descriptive differences were not used to infer model superiority or equivalence.

Stage 3, shuffling versus trembling within kinetic FoG, showed lower and more variable performance across held-out participant folds. The combined Logistic Regression yielded a mean balanced accuracy of 0.666±0.133, compared with 0.656±0.138 for the raw temporal CNN, 0.617±0.096 for Random Forest, and 0.601±0.098 for RBF-SVM. The CNN-minus-Logistic Regression difference in mean balanced accuracy was −0.010. Logistic Regression yielded a ROC-AUC of 0.784±0.146, while fold-to-fold variability remained substantial. Stage-wise confusion matrices for the raw temporal 1D-CNN are provided in Figure S1.

Taken together, the stage-wise benchmark showed different descriptive performance patterns across the three conditional stages. The CNN had a numerically higher mean balanced accuracy than the traditional models at Stage 1, whereas its mean balanced accuracy was close to that of Logistic Regression at Stages 2 and 3. These differences were interpreted descriptively only. These model comparisons were descriptive only; no hypothesis-test *p*-values or statistical-superiority claims were derived from the five outer folds. The stage-wise balanced-accuracy patterns across all four model families are summarized in Figure 3.

### 3.4. Mixed-Subject Nested LOSO Sensitivity Analysis

A separately defined mixed-subject nested leave-one-subject-out sensitivity analysis restricted evaluation to participants who exhibited both target classes (Table 3, Panel B). Because this sensitivity analysis used different participant subsets and a different resampling scheme from the main five-fold benchmark, its performance estimates were interpreted independently rather than as direct numerical comparisons with Panel A. All three feature representations were evaluated using Logistic Regression.

For Stage 2, eight participants exhibited both kinetic and akinetic FoG. Using the pre-specified combined nine-feature Logistic Regression, the subject-equal balanced accuracy was 0.634±0.110, with macro-F1 0.586±0.099 and ROC-AUC 0.673±0.143. The four-feature temporal and five-feature spectral subsets yielded balanced accuracies of 0.631±0.097 and 0.638±0.132, respectively. The small differences among feature sets, together with the between-participant variability, indicated that kinetic–akinetic discrimination remained heterogeneous when the held-out participant was required to contain both phenotypes.

The Stage 3 mixed-subject sensitivity analysis included only five participants because eligibility required both shuffling and trembling to be observed within the same individual. This restriction applied only to the nested LOSO sensitivity analysis; the main Stage 3 benchmark included 15 participants. In this restricted subset, the combined nine-feature Logistic Regression achieved a balanced accuracy of 0.598±0.142, whereas the four-feature temporal subset achieved 0.742±0.107 and the five-feature spectral subset 0.572±0.183. The temporal subset also yielded macro-F1 0.596±0.171 and ROC-AUC 0.742±0.207. Because these estimates were based on only five independent held-out participants, the comparatively higher temporal-feature balanced accuracy was treated as an exploratory mechanistic observation rather than as evidence for post hoc model selection. The result was therefore not used to replace the pre-specified combined-feature Logistic Regression in the primary Stage 3 benchmark.

### 3.5. Clinical Associations of Protocol-Observed Digital Biomarkers

The pre-specified participant-level clinical associations are summarized in Table 4. Protocol-observed locomotor FoG burden showed a positive association with FoG-Q (ρ=0.536, 95% bootstrap CI [0.136, 0.791], N=22) and a stronger positive association with MDS-UPDRS III (ρ=0.785, 95% bootstrap CI [0.567, 0.896], N=22). In contrast, the akinetic fraction of observed FoG showed a weaker and more uncertain association with FoG-Q (ρ=−0.206, 95% bootstrap CI [−0.749, 0.365], N=16). These three pre-specified clinical associations are visualized in Figure 4.

These findings indicate that, within the structured acquisition protocol, the overall amount of locomotor FoG observed across participants was more closely aligned with clinical severity measures than the proportion of FoG expressed as complete akinesia. The confidence interval for the akinetic-fraction association was wide and included both negative and positive values, providing no clear directional evidence in this cohort. No hypothesis-test *p*-values were calculated, and the bootstrap confidence intervals were interpreted as measures of estimation uncertainty rather than as dichotomous significance tests.

Locomotor observation time had a median of 3.095 min [IQR 1.841–3.659] and ranged from 1.188 to 7.016 min across participants. Locomotor exposure duration was positively associated with protocol-observed locomotor FoG burden, with a Spearman rho of 0.504 and a 95% bootstrap CI of [0.041, 0.821]. In the exposure-adjusted sensitivity analysis, the association between locomotor FoG burden and FoG-Q remained positive, with a partial Spearman rho of 0.578 and a 95% bootstrap CI of [0.225, 0.784]. The corresponding exposure-adjusted association with MDS-UPDRS III was also positive, with a partial Spearman rho of 0.716 and a 95% bootstrap CI of [0.331, 0.873]. Accordingly, the burden measure reflects the proportion of FoG observed during the available walking and turning periods of the structured protocol and should not be interpreted as an estimate of daily-life FoG prevalence. Exploratory associations involving additional clinical variables and manifestation–composition measures are reported in Table S2 and were not used to define the principal clinical conclusions.

## 4. Discussion

The present study examined wearable inertial phenotyping of FoG within a clinically aligned three-stage framework and yielded four main findings. First, right-ankle inertial signals captured clinically interpretable descriptive differences among FoG manifestations, most consistently for the contrast between kinetic FoG and complete akinesia, whereas shuffling–trembling differences were more heterogeneous. Second, the value of learned temporal representation depended on the classification stage: the raw temporal 1D-CNN achieved the highest mean balanced accuracy for Stage 1 FoG recognition, whereas no comparable advantage was observed for the subsequent phenotype discrimination stages. Third, kinetic–akinetic FoG discrimination was captured at least as well by the interpretable nine-feature Logistic Regression as by the CNN, consistent with the marked differences in residual movement amplitude and movement rate between these manifestations. Finally, shuffling–trembling discrimination was less stable across unseen participants and was supported by a much smaller number of independent mixed-phenotype participants.

The hierarchy was used as a clinically meaningful decomposition of the problem rather than as an end-to-end decision cascade. Each stage was evaluated independently on the clinically eligible ground-truth subset, so the reported results characterize the intrinsic subject-independent discriminability of each conditional decision rather than the cumulative performance of a propagated multi-stage classifier. This distinction is important because the three nodes represented substantially different learning problems. Locomotor FoG recognition benefited most clearly from a learned temporal representation, whereas the coarser kinetic–akinetic phenotype distinction was already well represented by a compact set of biomechanically interpretable features. The finer shuffling–trembling distinction, in contrast, showed greater between-participant variability and remains less certain. The participant-level clinical analysis added a complementary observation: the amount of locomotor FoG observed during the structured protocol was more closely aligned with clinical severity than the relative composition of FoG manifestations. Accordingly, the methodological contribution of the present work lies not in automated manifestation recognition per se, which has been demonstrated in previous wearable studies, but in evaluating successive phenotype distinctions as conditional subject-independent decisions and examining their biomechanical interpretability, representation requirements, and participant-level clinical relationships [17,18,19].

The biomechanical analysis helps interpret the conditional phenotype results. The most consistent distinction was observed between kinetic and akinetic FoG. In participants who exhibited both manifestations, kinetic FoG was characterized by greater acceleration variability, acceleration jerk, angular-velocity variability, and angular-acceleration dynamics than complete akinesia. This pattern is consistent with the clinical distinction between FoG with observable ineffective lower-limb movement and FoG without clinically observable lower-limb movement that underlies the updated International Consortium for Freezing of Gait framework [31]. It is also compatible with earlier phenomenological descriptions of FoG as a heterogeneous phenomenon comprising shuffling, trembling, and motor-block manifestations [5]. In the present signals, the kinetic–akinetic contrast was therefore expressed primarily as a difference in the amount and rate of residual ankle motion rather than as the presence or absence of a single spectral signature.

This point is particularly relevant to the classical 3–8 Hz “freeze band”. Increased lower-limb power in this frequency range has long been associated with FoG and forms the basis of the freeze index and related inertial measures [8,9]. High-frequency oscillations have also been shown to emerge in a distal-to-proximal pattern during freezing, supporting the value of ankle and shank measurements for capturing trembling-related dynamics [8]. However, in the present Stage 2 analysis, acceleration relative power in the 3–8 Hz band showed an almost null median kinetic-minus-akinetic contrast and inconsistent directions across participants, while the acceleration freeze index was similarly heterogeneous. This does not conflict with the established usefulness of freeze-band measures for FoG detection. The freeze index used here quantifies the ratio between 3–8 Hz power and 0.5–3 Hz power rather than the absolute amplitude of freeze-band activity. Consequently, a higher value may reflect increased 3–8 Hz oscillatory power, reduced low-frequency power, or both. In a phenotype comparison, this ratio can therefore behave differently from movement-amplitude measures, particularly when one state is characterized by very little overall movement, as in complete akinesia. In addition, the Stage 2 kinetic class pooled shuffling and trembling, which can express different temporal and spectral patterns. A uniform participant-level kinetic-minus-akinetic direction of the freeze index was therefore not expected. Recent work has similarly emphasized that the conventional freeze index is most directly sensitive to trembling FoG and can be less reliable for shuffling or akinetic presentations [36]. Because absolute low-frequency and freeze-band powers were not analyzed separately at the participant level for this contrast, the present data do not allow the contribution of numerator and denominator changes to be assigned definitively in individual participants.

The finer shuffling–trembling comparison was less uniform. Shuffling FoG is characterized clinically by ineffective, markedly shortened steps, whereas trembling FoG is dominated by fast lower-limb oscillatory movements; these distinctions are broadly consistent with both historical subtype descriptions and the updated kinetic-trembling versus kinetic-no-trembling terminology [5,31]. Earlier kinematic work has shown that the transition toward freezing can involve progressive stride-length reduction and abnormal cadence regulation, highlighting that FoG-related motor breakdown is not restricted to high-frequency trembling [37]. In the mixed-subject Stage 3 analysis, angular-velocity magnitude variability was higher during shuffling than trembling in all five participants, whereas the remaining temporal and spectral contrasts were not directionally consistent. Overall angular-velocity variability and narrow-band oscillatory power quantify different aspects of movement, so greater gyroscope variability during shuffling should not be interpreted as evidence of greater tremulous activity. Instead, the heterogeneous feature pattern raises the possibility that shuffling–trembling differentiation depends more on the temporal organization of ineffective ankle motion than on a single amplitude- or frequency-domain marker. This interpretation is compatible with the exploratory advantage of the temporal feature subset in the mixed-subject Stage 3 analysis, but the small number of participants prevents a stronger mechanistic conclusion.

The comparison between interpretable features and learned temporal representations further showed that increased model flexibility was not uniformly beneficial across the three conditional tasks. Within the present framework, Stage 1 served as the reference FoG-recognition node rather than as a new binary detection paradigm. The largest descriptive difference in mean balanced accuracy was observed at this stage, where the raw temporal 1D-CNN achieved a mean balanced accuracy of 0.877±0.034, compared with 0.845±0.032 for RBF-SVM. Deep-learning approaches have previously shown that temporal representations learned directly from wearable inertial signals can support FoG detection without requiring an exclusively handcrafted feature pipeline [12]. Conventional feature-based approaches have likewise achieved useful FoG recognition during structured provoking protocols [13]. In the present comparison, the CNN was evaluated not only against a linear classifier but also against RBF-SVM and Random Forest, both of which had lower mean Stage 1 balanced accuracy in this dataset. This numerical pattern is compatible with the learned representation capturing temporal information not fully summarized by the compact nine-feature set, although the five outer folds do not constitute independent experimental replicates and therefore do not support a formal claim of model superiority.

The picture changed once the analysis moved from FoG recognition to phenotype discrimination. At Stage 2, the interpretable nine-feature Logistic Regression yielded a mean balanced accuracy of 0.730, whereas the raw temporal CNN yielded 0.727, while neither RBF-SVM nor Random Forest provided a consistent advantage. This convergence is consistent with the biomechanical results: kinetic and akinetic FoG differed strongly in residual movement amplitude and movement rate, properties that were explicitly represented by acceleration variability, jerk, angular-velocity variability, and angular-acceleration features. Under these conditions, a compact biomechanically interpretable representation may already capture much of the discriminative information available in the right-ankle signal, limiting the incremental value of a more flexible learned temporal model. This finding should not be interpreted as evidence that deep learning is generally unnecessary for FoG phenotyping; rather, it indicates that the usefulness of representation learning depends on the specific clinical decision being modeled.

Stage 3 further illustrates this task dependence. The modest and variable subject-independent classification performance should be interpreted alongside, rather than as contradictory to, the descriptive biomechanical summaries. A group-level shift in a feature distribution, or even a consistent within-participant direction for a single feature, does not necessarily define a stable multivariate decision boundary across unseen participants.

In the main five-fold benchmark, none of the nonlinear or deep models improved descriptively on the combined Logistic Regression, and all approaches showed substantial fold-to-fold variability. Stage 3 was also the smallest primary classification dataset, comprising 290 windows from 15 participants, with 83 shuffling and 207 trembling windows. Among the five participants who exhibited both manifestations, angular-velocity magnitude variability was directionally consistent, whereas the remaining temporal and spectral feature contrasts showed heterogeneous within-participant directions. The limited number of participants, unequal class representation, and heterogeneous feature patterns are therefore plausible contributors to the limited stability of subject-independent shuffling–trembling discrimination.

At the same time, the temporal feature subset showed a numerically higher mean balanced accuracy in the nested LOSO analysis restricted to the five participants who exhibited both shuffling and trembling. Previous wearable studies have demonstrated the feasibility of automatically distinguishing clinically defined FoG manifestations, including shuffling, trembling, and akinetic presentations [17,18,19]. The present analysis addresses the complementary question of how reliably such finer distinctions generalize to unseen participants and which signal representations are most informative for each conditional decision. The present findings are compatible with a role for temporal organization in this finer distinction, but the small number of mixed participants prevents determining whether the observed Stage 3 pattern reflects a reproducible signal characteristic or cohort-specific variability.

More broadly, these findings highlight the distinction between predictive performance and transportability. Deep models can learn richer signal representations, but increased representational capacity does not by itself guarantee more stable generalization across individuals or datasets. Cross-dataset evaluations of FoG detection have reported substantial performance degradation despite competitive within-dataset results, underscoring the continuing challenge of robust generalization across cohorts, sensing protocols, and recording conditions [16]. In this context, interpretable biomechanical features retain practical value: they make the biomechanical basis of a decision more transparent, can reveal when a classical FoG marker is not informative for a particular phenotype distinction, and provide a meaningful benchmark against which the added value of learned representations can be judged. The present results therefore support a task-adaptive view rather than a single preferred model family: learned temporal representations were most useful for FoG recognition, whereas interpretable features remained competitive for conditional phenotype discrimination.

The mixed-subject sensitivity analyses emphasized that aggregate window-level performance does not fully characterize generalization across individuals. Restricting evaluation to participants who actually exhibited both target phenotypes created a more stringent test of within-participant phenotype discrimination. For Stage 2, the combined nine-feature Logistic Regression achieved a subject-equal balanced accuracy of 0.634±0.110 across the eight mixed participants, Because this sensitivity analysis used a different participant subset and resampling scheme from the main five-fold benchmark, its performance estimate was interpreted independently rather than as a direct increase or decrease relative to the primary analysis. Nevertheless, the nested LOSO result indicates that kinetic–akinetic discrimination remained moderate and heterogeneous when every held-out participant contributed both classes. Such inter-individual variability is a recurrent challenge in wearable FoG modeling, where person-specific manifestations and limited numbers of participants with sufficiently diverse FoG presentations constrain subject-independent generalization [14].

The same issue was even more pronounced for Stage 3. Only five participants exhibited both shuffling and trembling, substantially limiting the number of independent observations available to characterize generalization. Within this restricted group, the temporal feature subset yielded a higher mean balanced accuracy than the combined or spectral representations, but this result cannot establish a stable representation advantage. Rather, it should be viewed as an exploratory indication that the temporal organization of kinetic FoG may contain phenotype information whose expression varies across individuals. Larger cohorts in which both manifestations are repeatedly observed within the same participants will be necessary to determine whether this pattern is reproducible.

The participant-level clinical analysis further showed that protocol-observed locomotor FoG burden carried different information from manifestation composition. Locomotor FoG burden was positively associated with FoG-Q (ρ=0.536, 95% bootstrap CI [0.136, 0.791]) and MDS-UPDRS III (ρ=0.785, 95% bootstrap CI [0.567, 0.896]), whereas the akinetic fraction showed a weaker and imprecise association with FoG-Q (ρ=−0.206, 95% bootstrap CI [−0.749, 0.365]). This pattern suggests that, within the present structured protocol, the amount of locomotor freezing observed may be more closely related to overall clinical severity than the relative predominance of a particular FoG manifestation.

Because the burden measure was defined as the proportion of eligible locomotor samples annotated as FoG, longer observation did not mechanically increase the burden percentage. Nevertheless, unequal observation time can affect both the opportunity to observe an episodic phenomenon such as FoG and the precision of an individual burden estimate. Consistent with this concern, locomotor exposure duration was positively associated with the observed burden. However, rank-based adjustment for exposure duration did not eliminate the positive associations of burden with either FoG-Q or MDS-UPDRS III. These sensitivity results reduce the likelihood that the observed clinical associations were attributable solely to differences in locomotor exposure, but they do not remove the limitations of unequal and protocol-specific observation.

The finding is consistent with the broader concept that FoG severity cannot be fully represented by a single assessment modality. Wearable daily-living monitoring, structured FoG-provoking tests, and patient-reported measures capture overlapping but non-equivalent aspects of the disorder, and their relationships can vary according to assessment context [20].

This distinction is also relevant to the wider wearable-neurology literature. Wearable inertial sensing has the potential to complement conventional clinical assessment by providing objective measurements during functional activity and, in appropriate settings, continuous or unsupervised monitoring. At the same time, heterogeneity in sensor placement, gait metrics, acquisition protocols, and analytical procedures remains an important challenge for clinical interpretation and standardization [38]. Although that evidence extends beyond FoG specifically, it reinforces the need to distinguish what a wearable-derived quantity measures under a structured protocol from what it may represent during everyday behavior.

Accordingly, the present clinical correlations should not be interpreted as validation of a diagnostic biomarker or as estimates of free-living FoG prevalence. Locomotor observation time varied substantially across participants, and the digital burden measure was calculated only from walking and turning periods available within the structured acquisition. Participants in whom no FoG was observed during the protocol therefore contribute meaningful zero values to this protocol-specific burden measure, but they should not be considered clinical “non-freezers” on that basis alone. The current findings are better viewed as evidence that protocol-observed wearable quantities can show clinically coherent associations while remaining sensitive to the context and amount of observation.

Several limitations should be considered when interpreting these findings. First, the effective cohort became progressively smaller at the finer phenotype levels. Although the full cohort comprised 22 participants, only 16 contributed to Stage 2 and 15 to Stage 3, and the number of participants exhibiting both target manifestations decreased further in the mixed-subject analyses. In particular, only five participants exhibited both shuffling and trembling. This limits the diversity of subject-independent phenotype patterns available for training and evaluation and increases the uncertainty of Stage 3 estimates. The comparatively higher performance of the temporal feature subset in the Stage 3 nested LOSO analysis should therefore remain exploratory and should not be interpreted as justification for replacing the pre-specified combined-feature Logistic Regression after inspection of the results.

Second, the sensing and signal-processing configuration was intentionally narrow. All analyses used a single right-ankle IMU, so the findings do not establish whether the same phenotype information would be retained at alternative body locations, with bilateral sensing, or with reduced sensing modalities. The one-second analysis windows also imply an approximately 1 Hz frequency-bin spacing at the native 60 Hz sampling rate, providing only coarse spectral resolution for phenomena within the classical 3–8 Hz freeze band. Moreover, FoG manifestations were not uniformly distributed across activities and tasks. This issue was especially relevant to Stage 1 because FoG was uncommon during walking but substantially more frequent during turning. Nevertheless, when the frozen outer-test predictions were evaluated separately within walking, right-turning, and left-turning contexts, the CNN retained descriptive balanced accuracies of 0.839, 0.785, and 0.821, respectively. Thus, the Stage 1 result cannot be explained solely by a classifier distinguishing walking from turning. Although the stage definitions restricted the eligible clinical states and participant-independent splitting prevented direct subject overlap, residual activity- or task-related confounding cannot be excluded. In particular, the walking stratum contained relatively few FoG windows and only five participants contributed walking FoG, limiting the precision of that context-specific estimate. The classifiers may therefore have captured a mixture of manifestation-related and context-related signal characteristics.

Third, the recordings were obtained during a structured FoG-provoking protocol rather than during unconstrained daily life. Structured tasks are useful for eliciting observable FoG, but they do not reproduce the full variability of everyday walking environments, behavioral adaptation, day-to-day fluctuations, or spontaneous trigger conditions. In addition, locomotor observation time varied across participants. Although the burden was normalized by the amount of available locomotor observation and therefore was not mechanically inflated by longer recordings, unequal exposure may affect the opportunity to observe episodic FoG and the precision of participant-level estimates. The exposure-adjusted sensitivity analysis addressed this issue partially but cannot substitute for standardized or repeated observation periods. Consequently, protocol-observed locomotor FoG burden should not be interpreted as an estimate of daily-life FoG prevalence. Similarly, participants with a burden value of zero are more accurately described as having no FoG observed during the protocol; this designation does not establish that they are clinical “non-freezers”. The clinical correlations reported here are therefore context-specific associations and do not establish causality, diagnostic validity, or longitudinal responsiveness. Exploratory clinical associations should also be interpreted cautiously given the small number of participants and the absence of multiplicity-adjusted inferential testing.

Finally, the validation design supports subject-independent benchmarking but not external validation. The outer participant partitions were frozen and kept separate from preprocessing, weighting, hyperparameter selection, and early stopping within each analysis; however, the same dataset and frozen outer-fold structure were used repeatedly during model development and comparison. The resulting performance estimates should therefore be interpreted as subject-independent development benchmarks rather than as evaluation on an untouched external cohort. No independent cohort was available to assess transportability across centers, devices, protocols, or populations. In addition, the reported endpoints were calculated at the one-second window level. Successful window classification does not necessarily translate into accurate detection, onset localization, duration estimation, or phenotyping of complete FoG events, particularly when adjacent predictions must be combined over time. Independent event-level evaluation in external and naturalistic datasets will therefore be required before the proposed framework can be considered generalizable for continuous clinical monitoring.

The next priority is independent validation of the complete analytical framework in larger external cohorts. A first external evaluation should apply the locked preprocessing rules, feature definitions, model specifications, and decision rules without cohort-specific retuning; any subsequent recalibration or model adaptation should be evaluated separately. This would allow the present subject-independent development results to be distinguished from true transportability across recruitment settings, sensing systems, and clinical populations. Cross-dataset work in wearable FoG detection has already shown that performance can decrease substantially when models are transferred between datasets [16]; evaluating whether the present phenotype-oriented framework is similarly sensitive to dataset shift is therefore essential.

A second priority is to extend phenotype assessment from structured FoG-provoking protocols to longitudinal and free-living monitoring. Structured protocols remain valuable because they increase the probability of observing clinically relevant manifestations under controlled conditions, but everyday mobility introduces a broader range of walking contexts, behavioral adaptations, and trigger situations. Previous work comparing daily-living monitoring with structured FoG-provoking tests and self-report has shown that these assessment modalities provide related but non-equivalent information [20]. Future studies should therefore examine whether protocol-derived phenotype signatures remain identifiable during naturalistic behavior and whether wearable-derived FoG burden is reproducible across repeated days and recording contexts.

Event-level evaluation will also be necessary for clinical translation. The present work quantified discrimination in one-second windows, whereas practical monitoring requires reliable identification of complete FoG events, including onset, offset, duration, and manifestation composition. Future evaluations should consequently report event sensitivity together with false-alarm burden, temporal localization error, and agreement in event-level phenotype assignment. This is particularly important for episodes in which manifestations evolve over time, because transitions between shuffling, trembling, and akinesia may contain clinically relevant information that is not represented by an isolated window label.

Larger and more phenotype-balanced cohorts are especially important for the finer levels of the framework. Future acquisitions should aim to include substantially more participants who exhibit both kinetic and akinetic FoG and, in particular, both shuffling and trembling within the same individual. Repeated observations across tasks, sessions, and clinically relevant states would make it possible to separate stable participant-specific characteristics from context-dependent phenotype expression and to determine whether the temporal Stage 3 pattern observed here is reproducible. Such datasets would also support stratified analyses of task and activity context and reduce the risk that phenotype classifiers learn contextual cues specific to a particular acquisition protocol.

Finally, the sensing configuration should be evaluated more broadly before prospective implementation. The right ankle provided a consistent lower-limb signal in the present study, but alternative placements, bilateral configurations, and reduced sensor sets may offer different trade-offs between phenotype information and practical wearability. Future work should therefore compare sensor locations under identical participant-independent evaluation and determine the minimum sensing configuration that preserves clinically useful performance. Once externally validated, the selected models should be tested prospectively in a wearable implementation with direct measurement of latency, memory requirements, power consumption, and robustness to real-world signal quality. Together, these steps would determine whether clinically aligned FoG phenotyping can progress from a controlled analytical framework toward reliable longitudinal assessment in everyday neurological care.

## 5. Conclusions

Within this subject-independent development benchmark, right-ankle inertial signals showed clinically interpretable differences among FoG motor manifestations within a three-stage conditional phenotyping framework.

Kinetic–akinetic discrimination was associated most consistently with interpretable measures of residual movement amplitude and movement rate, whereas shuffling–trembling discrimination was weaker, more variable, and more heterogeneous across participants.

For locomotor FoG recognition, the raw temporal CNN yielded a numerically higher mean balanced accuracy than the traditional models, whereas the mean performance patterns at the two conditional phenotype-discrimination stages did not indicate a consistent descriptive advantage for the learned representation. These model comparisons were descriptive and do not establish statistical superiority or equivalence.

Protocol-observed locomotor FoG burden was positively associated with FoG-Q and MDS-UPDRS III, and these associations remained positive in the exposure-adjusted sensitivity analysis, whereas relationships involving manifestation composition were more uncertain.

The three stages were evaluated independently on their ground-truth eligible subsets and should therefore be interpreted as a clinically aligned decomposition of FoG phenotyping rather than as an end-to-end decision cascade.

Because all model development and evaluation were performed within the same dataset, the present results do not constitute external validation. Independent, phenotype-balanced cohorts together with event-level and free-living evaluation are required to determine transportability, reproducibility, and potential clinical utility.

## Figures and Tables

**Figure 1:**
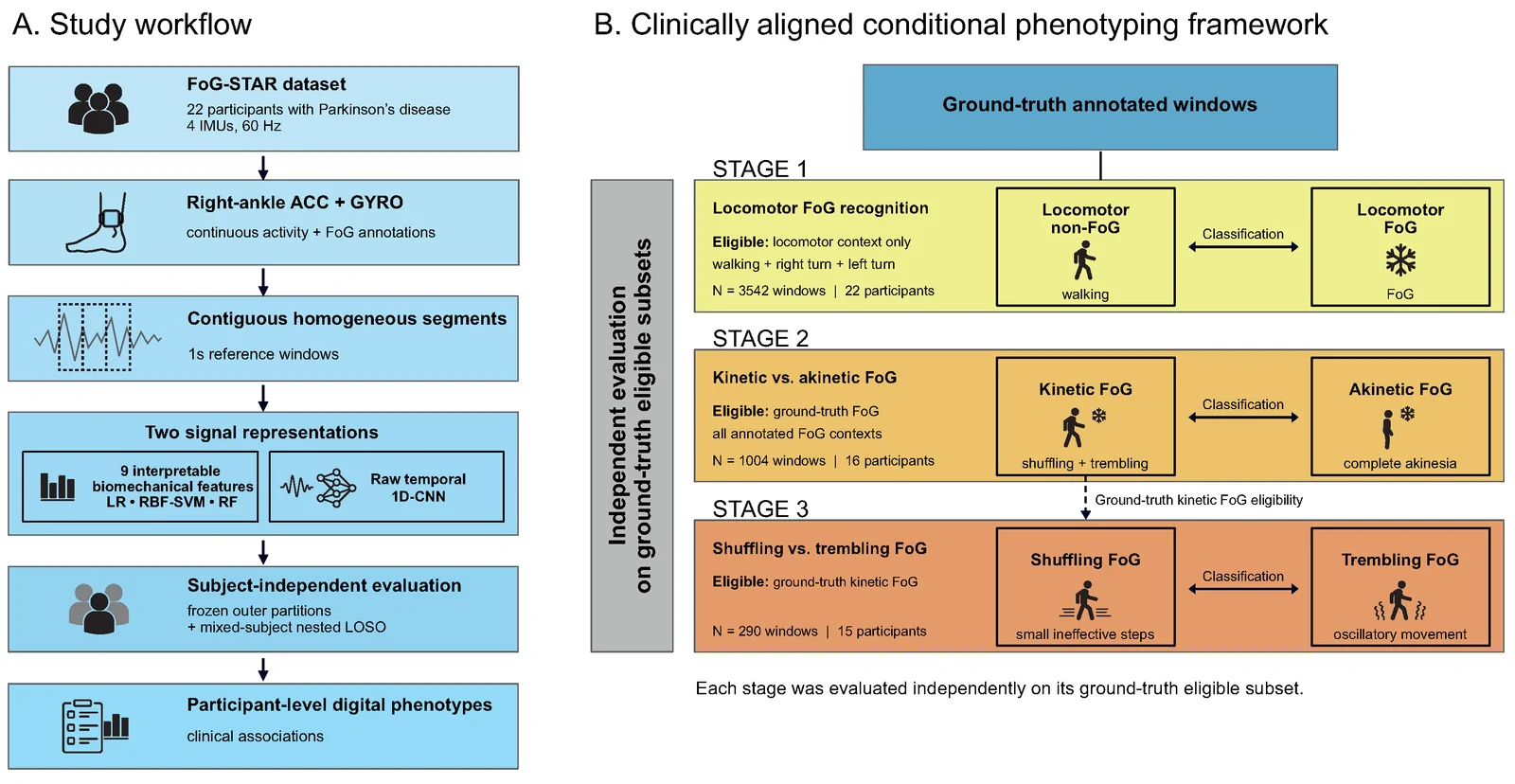
Study workflow and clinically aligned conditional FoG phenotyping framework. (**A**) Overview of the analytical workflow, from the FoG-STAR dataset and right-ankle accelerometer and gyroscope signals to 1 s reference windows, interpretable biomechanical features, raw temporal 1D-CNN representations, subject-independent evaluation, and participant-level digital phenotypes. (**B**) Three conditional classification stages evaluated independently on their clinically eligible ground-truth subsets. Stage 1 distinguishes locomotor non-FoG from locomotor FoG and is restricted to walking and turning contexts. Stage 2 distinguishes kinetic from akinetic FoG using all annotated ground-truth FoG contexts, with kinetic FoG comprising shuffling and trembling. Stage 3 distinguishes shuffling from trembling within ground-truth kinetic FoG. Predictions were not propagated between stages.

**Figure 2:**
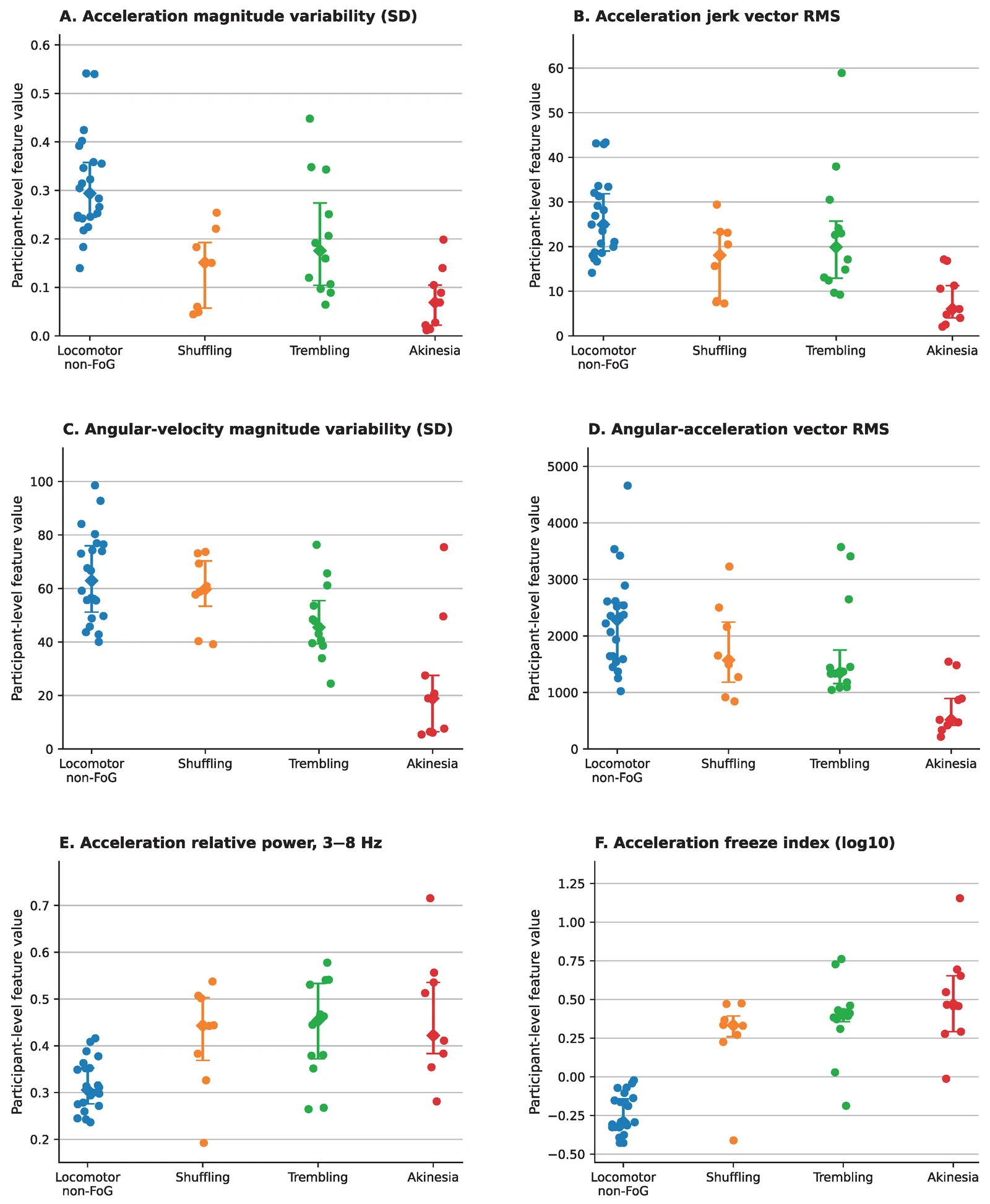
Selected subject-level biomechanical signatures across locomotor non-FoG, shuffling, trembling, and akinesia. (**A**) Acceleration magnitude variability; (**B**) acceleration jerk vector RMS; (**C**) angular-velocity magnitude variability; (**D**) angular-acceleration vector RMS; (**E**) acceleration relative power in the 3–8 Hz band; and (**F**) acceleration freeze index. Individual points represent participant-level feature summaries for each available motor state, with N=22 for locomotor non-FoG, N=8 for shuffling, N=12 for trembling, and N=9 for akinesia. Larger markers and error bars indicate the median and interquartile range across eligible participants. Panels (**A**–**D**) characterize movement-amplitude and movement-rate dynamics, whereas Panels (**E**,**F**) represent acceleration-based spectral measures related to the classical freeze band. The figure is descriptive; paired within-participant contrasts are reported in Table 2.

**Figure 3:**
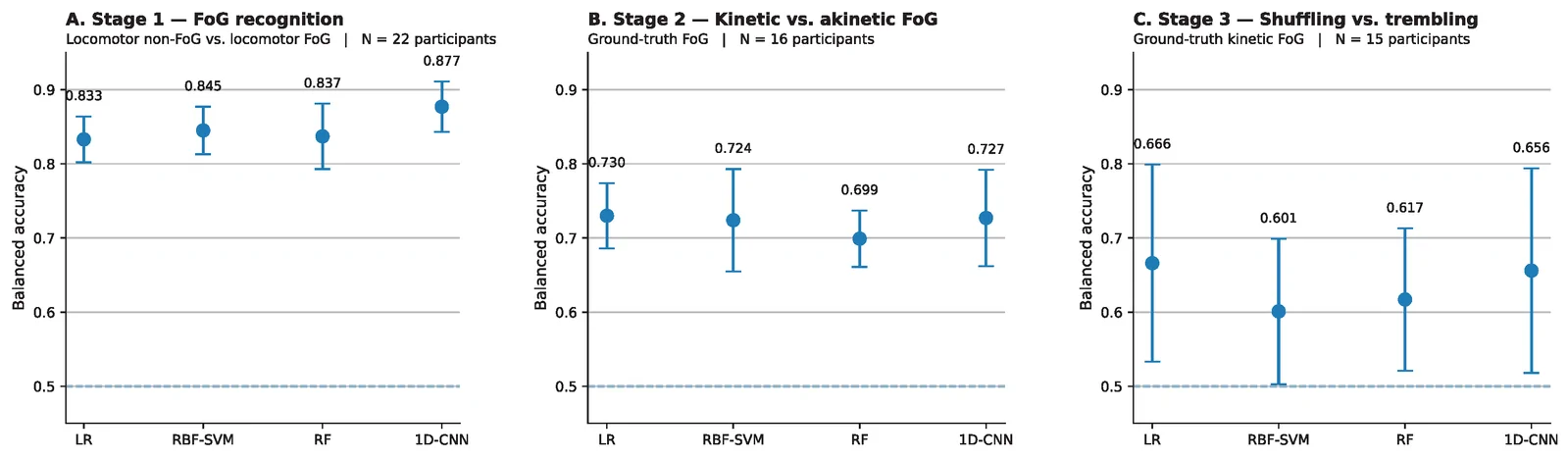
Subject-independent stage-wise classification performance. Balanced accuracy is shown for Logistic Regression (LR), RBF-SVM, Random Forest (RF), and the raw temporal 1D-CNN across the three conditional classification stages: (**A**) locomotor non-FoG versus locomotor FoG; (**B**) kinetic versus akinetic FoG; and (**C**) shuffling versus trembling. Points indicate mean balanced accuracy and error bars indicate standard deviation across the five frozen participant-independent outer folds. The horizontal dashed line denotes the balanced-accuracy chance level of 0.50. Model comparisons are descriptive; no statistical-superiority tests were performed.

**Figure 4:**
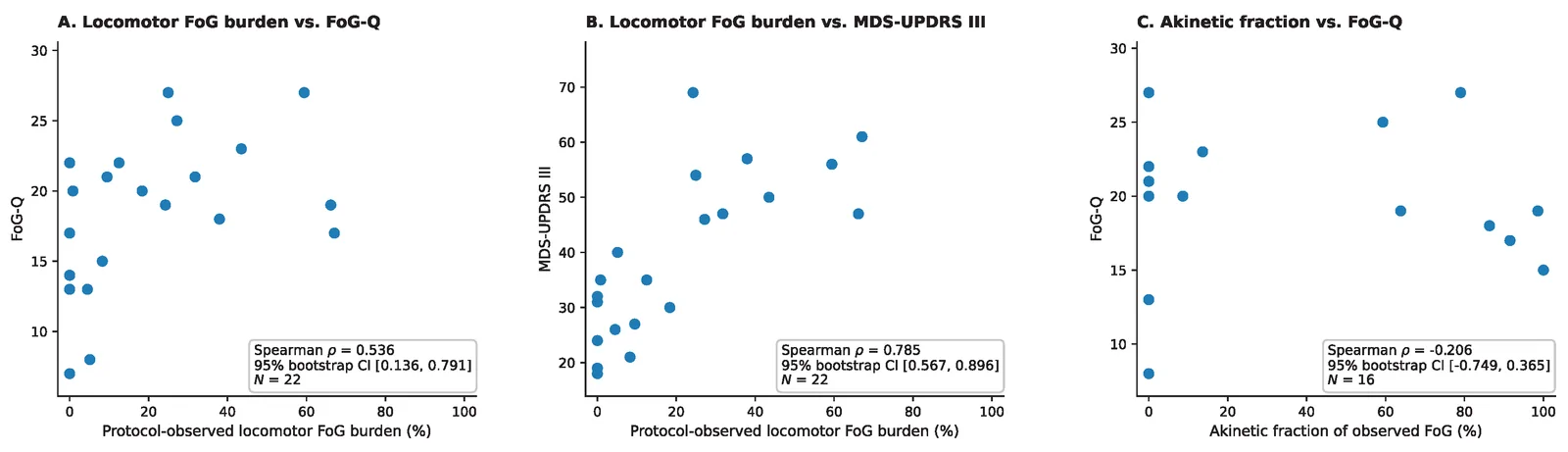
Associations between protocol-observed digital FoG measures and clinical severity. (**A**) Protocol-observed locomotor FoG burden versus FoG-Q (ρ=0.536, 95% bootstrap CI [0.136, 0.791], N=22); (**B**) protocol-observed locomotor FoG burden versus MDS-UPDRS III (ρ=0.785, 95% bootstrap CI [0.567, 0.896], N=22); and (**C**) akinetic fraction of observed FoG versus FoG-Q (ρ=−0.206, 95% bootstrap CI [−0.749, 0.365], N=16). Each point represents one participant. Associations were quantified using Spearman rank correlation, with 95% confidence intervals obtained by participant-level bootstrap resampling. No hypothesis-test *p*-values were calculated. Locomotor FoG burden reflects FoG observed during walking and turning periods of the structured protocol and should not be interpreted as daily-life FoG prevalence.

**Table 1:** Participant characteristics. Continuous variables are reported as median [interquartile range]; sex is reported as n/N (%).

Characteristic	Overall	FoG Observed During Protocol	No FoG Observed During Protocol
Female sex, n/N (%)	10/22 (45.5%)	9/16 (56.2%)	1/6 (16.7%)
Age, years	71.00 [65.00, 76.00]	71.00 [65.00, 76.00]	71.00 [63.75, 79.75]
Disease duration, years	9.00 [4.00, 15.00]	9.00 [5.00, 11.50]	11.50 [3.00, 17.00]
Hoehn and Yahr stage	2.50 [2.00, 3.00]	3.00 [2.25, 4.00]	2.00 [2.00, 2.00]
MDS-UPDRS III	35.00 [27.75, 49.25]	46.50 [33.75, 54.50]	27.50 [20.25, 31.75]
FoG-Q	19.00 [14.25, 21.75]	20.00 [17.75, 22.25]	14.00 [13.25, 16.25]
MoCA	22.00 [19.00, 24.00]	21.00 [17.00, 22.50]	24.00 [21.00, 24.00]
FES-I	27.00 [18.00, 53.00]	27.00 [20.50, 67.50]	19.00 [13.75, 29.50]
PDQ-8	14.00 [10.00, 18.25]	15.50 [12.75, 21.25]	9.00 [6.00, 10.50]

*Note*: Disease duration, Hoehn and Yahr stage, MoCA, and FES-I were available for 21 participants overall and for 15 of the 16 participants with FoG observed during the protocol; all other variables were complete. FoG = freezing of gait; MDS-UPDRS III = Movement Disorder Society-sponsored revision of the Unified Parkinson’s Disease Rating Scale, Part III; FoG-Q = Freezing of Gait Questionnaire; MoCA = Montreal Cognitive Assessment; FES-I = Falls Efficacy Scale–International; PDQ-8 = Parkinson’s Disease Questionnaire–8.

**Table 2:** Selected subject-level biomechanical signatures across motor states and paired within-participant phenotype contrasts. State values are median [interquartile range] of participant-level medians. Paired contrasts are reported as the median within-participant difference followed by the number of participants with positive and negative differences.

Feature	Locomotor Non-FoG	Shuffling	Trembling	Akinesia	Stage 2: Kinetic–Akinetic	Stage 3: Shuffling–Trembling
Acceleration magnitude variability (SD), g	0.294 [0.244, 0.358]	0.151 [0.057, 0.192]	0.176 [0.104, 0.274]	0.069 [0.022, 0.105]	0.114; +7/8, −1/8	−0.071; +1/5, −4/5
Acceleration jerk vector RMS, g/s	24.940 [19.012, 31.834]	18.060 [7.744, 23.153]	19.870 [12.920, 25.701]	6.011 [4.011, 11.262]	9.888; +8/8, −0/8	−5.823; +2/5, −3/5
Angular-velocity magnitude variability (SD), deg/s	62.920 [51.154, 75.959]	59.833 [53.350, 70.288]	45.462 [39.315, 55.467]	18.925 [6.455, 27.479]	18.585; +7/8, −1/8	12.009; +5/5, −0/5
Angular-acceleration vector RMS, deg/s^2^	2283.953 [1602.345, 2592.486]	1572.330 [1180.805, 2244.754]	1350.009 [1157.011, 1750.055]	515.614 [418.221, 891.970]	574.972; +7/8, −1/8	−61.559; +2/5, −3/5
Acceleration relative power, 3–8 Hz	0.305 [0.276, 0.352]	0.443 [0.369, 0.503]	0.454 [0.372, 0.533]	0.422 [0.383, 0.535]	0.002; +4/8, −4/8	0.044; +3/5, −2/5
Acceleration freeze index ($\log_{10}$)	−0.293 [−0.326, −0.141]	0.333 [0.260, 0.393]	0.402 [0.356, 0.438]	0.466 [0.292, 0.653]	−0.101; +3/8, −5/8	−0.085; +2/5, −3/5

*Note*: Stage 2 paired contrasts include the 8 participants exhibiting both kinetic and akinetic FoG. Stage 3 paired contrasts include the 5 participants exhibiting both shuffling and trembling. These summaries are descriptive; no hypothesis-test *p*-values were calculated. RMS = root mean square; SD = standard deviation; FoG = freezing of gait.

**Table 3:** Subject-independent classification performance and mixed-subject sensitivity analysis. For the main benchmark (Panel A), values are mean ± standard deviation across the five frozen participant-independent outer folds. For the nested LOSO sensitivity analysis (Panel B), values are mean ± standard deviation across held-out mixed participants. Balanced accuracy was the primary metric; macro-F1 and ROC-AUC were secondary metrics. Model comparisons are descriptive.

Analysis	Stage	Model/Feature Set	Subjects	Balanced Accuracy	Macro-F1	ROC-AUC
*Panel A. Main five-fold subject-independent benchmark*						
Main benchmark	Stage 1	Logistic Regression	22	0.833±0.031	0.790±0.042	0.912±0.035
Main benchmark	Stage 1	RBF-SVM	22	0.845±0.032	0.799±0.029	0.911±0.033
Main benchmark	Stage 1	Random Forest	22	0.837±0.044	0.821±0.042	0.925±0.029
Main benchmark	Stage 1	Raw temporal 1D-CNN	22	0.877±0.034	0.835±0.049	0.934±0.032
Main benchmark	Stage 2	Logistic Regression	16	0.730±0.044	0.720±0.057	0.816±0.053
Main benchmark	Stage 2	RBF-SVM	16	0.724±0.069	0.706±0.088	0.790±0.075
Main benchmark	Stage 2	Random Forest	16	0.699±0.038	0.681±0.047	0.788±0.044
Main benchmark	Stage 2	Raw temporal 1D-CNN	16	0.727±0.065	0.684±0.048	0.825±0.053
Main benchmark	Stage 3	Logistic Regression	15	0.666±0.133	0.635±0.134	0.784±0.146
Main benchmark	Stage 3	RBF-SVM	15	0.601±0.098	0.545±0.104	0.696±0.080
Main benchmark	Stage 3	Random Forest	15	0.617±0.096	0.586±0.116	0.701±0.126
Main benchmark	Stage 3	Raw temporal 1D-CNN	15	0.656±0.138	0.589±0.171	0.713±0.149
*Panel B. Mixed-subject nested LOSO sensitivity analysis*						
Nested LOSO	Stage 2	Combined (9 features)	8	0.634±0.110	0.586±0.099	0.673±0.143
Nested LOSO	Stage 2	Temporal (4 features)	8	0.631±0.097	0.593±0.091	0.686±0.167
Nested LOSO	Stage 2	Spectral (5 features)	8	0.638±0.132	0.516±0.133	0.651±0.155
Nested LOSO	Stage 3	Combined (9 features)	5	0.598±0.142	0.483±0.060	0.734±0.061
Nested LOSO	Stage 3	Temporal (4 features)	5	0.742±0.107	0.596±0.171	0.742±0.207
Nested LOSO	Stage 3	Spectral (5 features)	5	0.572±0.183	0.452±0.089	0.596±0.183

*Note*: Stage 1 = locomotor non-FoG versus locomotor FoG; Stage 2 = kinetic versus akinetic FoG conditional on ground-truth FoG; Stage 3 = shuffling versus trembling conditional on ground-truth kinetic FoG. LOSO = leave-one-subject-out; RBF-SVM = radial-basis-function support-vector machine; CNN = convolutional neural network; ROC-AUC = area under the receiver-operating-characteristic curve. All Panel B entries used L2-regularized Logistic Regression. The feature representation is identified explicitly as combined (9 features), temporal (4 features), or spectral (5 features). Panel B represents a separately defined sensitivity analysis and should not be interpreted as a direct numerical comparison with Panel A because the participant subsets and resampling schemes differ.

**Table 4:** Pre-specified primary associations between protocol-observed digital FoG biomarkers and clinical measures. Spearman rank-correlation coefficients are reported with 95% participant-level bootstrap confidence intervals.

Digital Biomarker	Clinical Variable	Analysis Set	*N*	Spearman ρ [95% CI]
Protocol-observed locomotor FoG burden (%)	FoG-Q	All participants	22	0.536 [0.136, 0.791]
Protocol-observed locomotor FoG burden (%)	MDS-UPDRS III	All participants	22	0.785 [0.567, 0.896]
Akinetic fraction of observed FoG (%)	FoG-Q	Participants with protocol-observed FoG	16	−0.206 [−0.749, 0.365]

*Note*: Confidence intervals were obtained by participant-level nonparametric bootstrap resampling. No hypothesis-test *p*-values were calculated. FoG = freezing of gait; FoG-Q = Freezing of Gait Questionnaire; MDS-UPDRS III = Movement Disorder Society-sponsored revision of the Unified Parkinson’s Disease Rating Scale, Part III.

## Data Availability

The data analyzed in this study are publicly available in the FoG-STAR repository on Zenodo (doi:10.5281/zenodo.17838806) under the Creative Commons Attribution 4.0 International license. The dataset includes the inertial-sensor recordings, FoG and activity annotations, and participant-level demographic and clinical variables used in the present analyses. The analysis code, frozen analysis configurations, and derived non-sensitive outputs supporting the results of this study are publicly available at https://github.com/augustoielo/wearable-fog-phenotyping (accessed on 12 September 2026). The repository includes the participant-independent split definitions and the code and fixed analysis specifications required to reproduce the feature-based analyses, CNN training and seed ensembling, sensitivity analyses, and derived manuscript-level outputs.

## Supplementary Materials

The following supporting information can be downloaded at https://www.mdpi.com/article/10.3390/bioengineering13101150/s1: Figure S1: Stage-wise CNN confusion matrices; Table S1: Full nine-feature biomechanical summary; Table S2: Exploratory clinical associations; Table S3: Stage-1 activity-context sensitivity analysis; Table S4: Participant-level window counts by classification stage.

## Abbreviations

The following abbreviations are used in this manuscript:

ACC	Accelerometer/acceleration signal
BA	Balanced accuracy
CNN	Convolutional neural network
FES-I	Falls Efficacy Scale–International
FFT	Fast Fourier transform
FoG	Freezing of gait
FoG-Q	Freezing of Gait Questionnaire
GYRO	Gyroscope/angular-velocity signal
IMU	Inertial measurement unit
LOSO	Leave-one-subject-out
MDS-UPDRS III	Movement Disorder Society Unified Parkinson’s Disease Rating Scale, Part III
MoCA	Montreal Cognitive Assessment
PD	Parkinson’s disease
PDQ-8	Parkinson’s Disease Questionnaire–8
RF	Random forest
RMS	Root mean square
ROC-AUC	Area under the receiver operating characteristic curve
SVM	Support vector machine
