# Supplementary material for: Wearable Inertial Phenotyping of Freezing of Gait in Parkinson’s Disease: Biomechanical Signatures, Subject-Independent Classification, and Clinical Associations
Source: Bioengineering (Basel). 2026 Oct 2;13(10):1150. doi: 10.3390/bioengineering13101150 (PMC13701957; doi:10.3390/bioengineering13101150)
Supplement: Supplementary file 1 [file bioengineering-13-01150-s001.zip › bioengineering-4593747-supplementary.pdf]

Supplementary Materials

Wearable Inertial Phenotyping of Freezing of Gait in Parkinson's Disease: Biomechanical Signatures, Subject-Independent Classification, and Clinical Associations

Augusto Ielo, Lilla Bonanno [*], Angelo Quartarone and Serena Dattola

IRCCS Centro Neurolesi Bonino-Pulejo, S.S. 113 Via Palermo, C. da Casazza, 98124 Messina, Italy; augusto.ielo@irccsme.it (A.I.); angelo.quartarone@irccsme.it (A.Q.); serena.dattola@irccsme.it (S.D.)

[*] Correspondence: lilla.bonanno@irccsme.it

Figure S1. Stage-Wise CNN Confusion Matrices

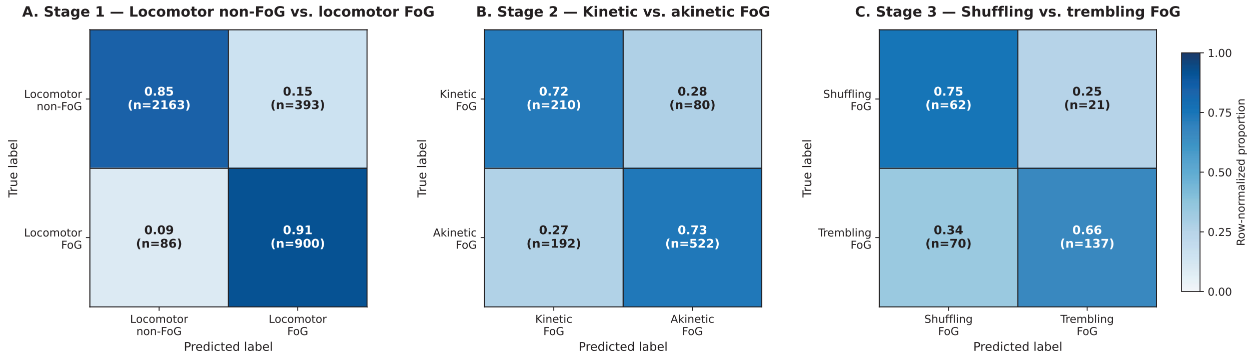

Figure S1: **Stage-wise confusion matrices for the raw temporal 1D-CNN.** (A) Stage 1, loco-motor non-FoG versus locomotor FoG; (B) Stage 2, kinetic versus akinetic FoG; and (C) Stage 3, shuffling versus trembling. Confusion matrices were calculated from seed-ensemble predictions on the frozen participant-independent outer-test folds, pooled across the five folds. Rows represent ground-truth classes and columns represent predicted classes. Cell shading and decimal values indi-cate row-normalized proportions, whereas values in parentheses indicate the corresponding number of outer-test windows. Each stage was evaluated independently on its clinically eligible ground-truth subset.

Table S1. Full Nine-Feature Biomechanical Summary

Table S1: **Full nine-feature biomechanical summary.** State values are participant-level medians [Q1, Q3]. Stage 2 contrasts are within-participant kinetic-minus-akinetic median differences among the eight participants exhibiting both states. Stage 3 contrasts are within-participant shuffling-minus-trembling median differences among the five participants exhibiting both kinetic manifestations. Direction counts indicate the number of participants with positive and negative paired differences.

**Feature**	**Locomotor non-FoG**	**Shuffling**	**Trembling**	**Akinesia**	**Stage 2: kinetic − akinetic**	**Stage 3: shuffling − trembling**
Acceleration magnitude variability (SD)	0.294 [0.244, 0.358]	0.151 [0.057, 0.192]	0.176 [0.104, 0.274]	0.069 [0.022, 0.105]	0.114; +7/8, −1/8	−0.071; +1/5, −4/5
Acceleration jerk vector RMS	24.940 [19.012, 31.834]	18.060 [7.744, 23.153]	19.870 [12.920, 25.701]	6.011 [4.011, 11.262]	9.888; +8/8, −0/8	−5.823; +2/5, −3/5
Angular-velocity magnitude variability (SD)	62.920 [51.154, 75.959]	59.833 [53.350, 70.288]	45.462 [39.315, 55.467]	18.925 [6.455, 27.479]	18.585; +7/8, −1/8	12.009; +5/5, −0/5
Angular-acceleration vector RMS	2283.953 [1602.345, 2592.486]	1572.330 [1180.805, 2244.754]	1350.009 [1157.011, 1750.055]	515.614 [418.221, 891.970]	574.972; +7/8, −1/8	−61.559; +2/5, −3/5
Acceleration relative power, 3–8 Hz	0.305 [0.276, 0.352]	0.443 [0.369, 0.503]	0.454 [0.372, 0.533]	0.422 [0.383, 0.535]	0.002; +4/8, −4/8	0.044; +3/5, −2/5
Acceleration freeze index ($\log_{10}$)	−0.293 [−0.326, −0.141]	0.333 [0.260, 0.393]	0.402 [0.356, 0.438]	0.466 [0.292, 0.653]	−0.101; +3/8, −5/8	−0.085; +2/5, −3/5
Acceleration spectral entropy	0.618 [0.606, 0.652]	0.865 [0.816, 0.880]	0.848 [0.804, 0.910]	0.831 [0.825, 0.844]	0.047; +5/8, −3/8	−0.013; +1/5, −4/5
Gyroscope relative power, 3–8 Hz	0.201 [0.164, 0.236]	0.212 [0.147, 0.229]	0.169 [0.148, 0.264]	0.249 [0.210, 0.267]	−0.065; +1/8, −7/8	−0.085; +1/5, −4/5
Gyroscope spectral entropy	0.495 [0.469, 0.514]	0.490 [0.473, 0.570]	0.503 [0.468, 0.585]	0.601 [0.583, 0.673]	−0.116; +1/8, −7/8	0.031; +3/5, −2/5

Table S2. Exploratory Clinical Associations

Table S2: **Exploratory participant-level clinical associations.** Spearman rank-correlation co-efficients are reported with 95% participant-level bootstrap confidence intervals based on 10,000 resamples. These analyses were exploratory; no hypothesis-test p-values were calculated and no multiplicity correction was applied.

**Digital biomarker**	**Clinical variable**	N	**Spearman ρ [95% bootstrap CI]**
Protocol-observed locomotor FoG burden (%)	Hoehn and Yahr stage	21	0.813 [0.552, 0.941]
Protocol-observed locomotor FoG burden (%)	FES-I	21	0.503 [0.110, 0.784]
Protocol-observed locomotor FoG burden (%)	PDQ-8	22	0.774 [0.492, 0.915]
Protocol-observed locomotor FoG burden (%)	Disease duration	21	0.214 [−0.291, 0.681]
Protocol-observed locomotor FoG burden (%)	MoCA	21	−0.467 [−0.763, −0.009]
Akinetic fraction of observed FoG (%)	MDS-UPDRS III	16	0.355 [−0.245, 0.819]
Akinetic fraction of observed FoG (%)	Hoehn and Yahr stage	15	0.399 [−0.214, 0.885]
Akinetic fraction of observed FoG (%)	FES-I	15	−0.089 [−0.623, 0.482]
Akinetic fraction of observed FoG (%)	PDQ-8	16	0.422 [−0.108, 0.848]
Akinetic fraction of observed FoG (%)	Disease duration	15	0.626 [0.166, 0.905]
Akinetic fraction of observed FoG (%)	MoCA	15	−0.238 [−0.751, 0.364]
Trembling fraction of kinetic FoG (%)	FoG-Q	15	0.512 [−0.031, 0.890]
Trembling fraction of kinetic FoG (%)	MDS-UPDRS III	15	0.363 [−0.179, 0.789]
Trembling fraction of kinetic FoG (%)	Hoehn and Yahr stage	14	0.449 [−0.141, 0.922]
Trembling fraction of kinetic FoG (%)	FES-I	14	0.404 [−0.175, 0.824]
Trembling fraction of kinetic FoG (%)	PDQ-8	15	0.315 [−0.290, 0.790]
Trembling fraction of kinetic FoG (%)	Disease duration	14	0.017 [−0.626, 0.602]
Trembling fraction of kinetic FoG (%)	MoCA	14	−0.175 [−0.695, 0.451]
Normalized manifestation entropy	FoG-Q	16	0.420 [−0.102, 0.818]
Normalized manifestation entropy	MDS-UPDRS III	16	0.281 [−0.260, 0.705]
Normalized manifestation entropy	Hoehn and Yahr stage	15	0.233 [−0.476, 0.718]
Normalized manifestation entropy	FES-I	15	0.170 [−0.487, 0.661]
Normalized manifestation entropy	PDQ-8	16	0.421 [−0.042, 0.759]
Normalized manifestation entropy	Disease duration	15	0.532 [0.002, 0.875]

**Digital biomarker**	**Clinical variable**	N	**Spearman ρ [95% bootstrap CI]**
Normalized manifestation entropy	MoCA	15	0.033 [−0.505, 0.487]

Note. Sample sizes reflect available-case analysis and the eligibility population for each digital biomarker. FoG = freezing of gait; FoG-Q = Freezing of Gait Questionnaire; MDS-UPDRS III = Movement Disorder Society-sponsored revision of the Unified Parkinson's Disease Rating Scale, Part III; FES-I = Falls Efficacy Scale–International; PDQ-8 = Parkinson's Disease Questionnaire-8; MoCA = Montreal Cognitive Assessment.

Table S3. Stage-1 Activity-Context Sensitivity Analysis

Table S3: **Stage 1 activity-context sensitivity analysis using frozen outer-test predictions.** Stage 1 predictions were stratified according to the ground-truth locomotor activity context as walking, right turning, or left turning. No classifier was retrained or retuned for this analysis, and activity labels were used only for post-prediction stratification and were not provided as classifier inputs. Metrics were calculated from the frozen participant-independent outer-test predictions pooled across the five outer folds. Balanced accuracy and ROC-AUC are reported only for activity strata containing both locomotor non-FoG and FoG windows.

**Model**	**Activity**	*N*	**Non-FoG**	**FoG**	**Balanced accuracy**	**Non-FoG recall**	**FoG recall**	**ROC-AUC**
Logistic Regression	Walking	1873	1817	56	0.831	0.876	0.786	0.883
Logistic Regression	Right turning	702	313	389	0.732	0.518	0.946	0.836
Logistic Regression	Left turning	967	426	541	0.766	0.678	0.854	0.847
RBF-SVM	Walking	1873	1817	56	0.834	0.882	0.786	0.883
RBF-SVM	Right turning	702	313	389	0.730	0.530	0.931	0.817
RBF-SVM	Left turning	967	426	541	0.775	0.655	0.895	0.837
Random Forest	Walking	1873	1817	56	0.839	0.928	0.750	0.902
Random Forest	Right turning	702	313	389	0.783	0.687	0.879	0.856
Random Forest	Left turning	967	426	541	0.789	0.784	0.793	0.877
Raw temporal 1D-CNN	Walking	1873	1817	56	0.839	0.910	0.768	0.886
Raw temporal 1D-CNN	Right turning	702	313	389	0.785	0.655	0.915	0.815
Raw temporal 1D-CNN	Left turning	967	426	541	0.821	0.716	0.926	0.869

Note. The activity distribution was markedly imbalanced with respect to FoG status: walking contained 1817 locomotor non-FoG and 56 FoG windows, whereas right turning contained 313 non-FoG and 389 FoG windows and left turning contained 426 non-FoG and 541 FoG windows. Nevertheless, all four model families retained descriptive discrimination when evaluated within each activity context. The activity-equal balanced accuracy, calculated as the unweighted mean of the three within-activity balanced accuracies, was 0.776 for Logistic Regression, 0.780 for RBF-SVM, 0.803 for Random Forest, and 0.815 for the raw temporal 1D-CNN. The walking stratum should be interpreted cautiously because only 56 FoG windows from five participants were available, and both classes were represented within the walking stratum in only three of the five outer folds. These analyses are descriptive and were not used for model retraining, hyperparameter selection, or threshold adjustment. FoG = freezing of gait; RBF-SVM = radial-basis-function support vector machine; ROC-AUC = area under the receiver-operating-characteristic curve.

Table S4. Participant-Level Window Counts by Classification Stage

Table S4: **Participant-level deterministic reference-window counts for the three classification stages.** Counts represent the frozen non-overlapping 1-s deterministic reference windows defining each stage-specific analysis set. Stage 1 was restricted to walking, right-turning, and left-turning contexts and contrasted locomotor non-FoG with locomotor FoG. Stage 2 included all annotated FoG contexts and contrasted kinetic FoG (shuffling plus trembling) with akinetic FoG. Stage 3 included the ground-truth kinetic-FoG subset and contrasted shuffling with trembling. A value of zero indicates that the corresponding class was not represented for that participant in the frozen stage-specific reference set.

**Participant**	**Stage 1**		**Stage 2**		**Stage 3**	
	**Locomotor non-FoG**	**FoG**	**Kinetic**	**Akinetic**	**Shuffling**	**Trembling**
1	83	173	2	171	2	0
2	132	274	24	267	0	24
3	132	84	11	73	0	11
4	188	10	10	0	10	0
5	55	5	0	5	0	0
6	59	0	0	0	0	0
7	146	0	0	0	0	0
8	59	0	0	0	0	0
9	165	32	30	2	29	1
10	198	0	0	0	0	0
11	141	51	20	31	0	20
12	151	49	16	33	15	1
13	131	18	18	0	5	13
14	85	0	0	0	0	0
15	117	54	54	0	0	54
16	112	1	1	0	0	1
17	62	3	3	0	3	0
18	171	0	0	0	0	0
19	103	157	32	126	0	32
20	149	14	14	0	5	9
21	52	40	34	6	14	20
22	65	21	21	0	0	21
**Total**	**2556**	**986**	**290**	**714**	**83**	**207**

Note. Stage-specific eligibility followed the same frozen definitions used in the primary analyses. Consequently, participant-level counts should not be summed across stages as independent observations: Stage 3 is the kinetic subset of Stage 2, whereas Stage 1 applies an additional locomotor-context restriction. The aggregate counts were 2556 locomotor non-FoG and 986 FoG windows for Stage 1; 290 kinetic and 714 akinetic FoG windows for Stage 2; and 83 shuffling and 207 trembling windows for Stage 3. FoG = freezing of gait.
